# Detection bias in open-label trials of anticancer drugs: a meta-epidemiological study

**DOI:** 10.1136/bmjebm-2023-112332

**Published:** 2023-08-16

**Authors:** Satoshi Funada, Yan Luo, Yuki Kataoka, Takashi Yoshioka, Yusuke Fujita, Shinya Yoshida, Morihiro Katsura, Masafumi Tada, Norihiro Nishioka, Yoshiaki Nakamura, Kentaro Ueno, Ryuji Uozumi, Toshi A Furukawa

**Affiliations:** 1 Department of Health Promotion and Human Behavior, Kyoto University Graduate School of Medicine and Faculty of Medicine / School of Public Health, Kyoto, Japan; 2 Department of Preventive Medicine and Public Health, Keio University School of Medicine, Tokyo, Japan; 3 Department of Internal Medicine, Kyoto Min-iren Asukai Hospital, Kyoto, Japan; 4 Section of Clinical Epidemiology, Department of Community Medicine, Kyoto University Graduate School of Medicine and Faculty of Medicine, Kyoto, Japan; 5 Department of Healthcare Epidemiology, Kyoto University Graduate School of Medicine and Faculty of Medicine / School of Public Health, Kyoto, Japan; 6 Scientific Research Works Peer Support Group (SRWS-PSG), Osaka, Japan; 7 Department of Surgery, Kyoto University Graduate School of Medicine and Faculty of Medicine, Kyoto, Japan; 8 Department of Surgery, Osaka Red Cross Hospital, Osaka, Japan; 9 Department of Surgery, Okinawa Chubu Hospital, Okinawa, Japan; 10 Human Health Science, Kyoto University Graduate School of Medicine and Faculty of Medicine, Kyoto, Japan; 11 Department of Neurology, Emergency Medicine, Nagoya City University East Medical Center, Nagoya, Japan; 12 Department of Preventive Services, Kyoto University Graduate School of Medicine and Faculty of Medicine / School of Public Health, Kyoto, Japan; 13 Department of Gastroenterology and Gastrointestinal Oncology, National Cancer Center-Hospital East, Kashiwa, Japan; 14 Translational Research Support Section, National Cancer Center Hospital East, Kashiwa, Japan; 15 Department of Biomedical Statistics and Bioinformatics, Kyoto University Graduate School of Medicine and Faculty of Medicine, Kyoto, Japan; 16 Department of Industrial Engineering and Economics, Tokyo Institute of Technology, Tokyo, Japan

**Keywords:** medical oncology

## Abstract

**Objectives:**

In anticancer clinical trials, particularly open-label trials, central reviewers are recommended to evaluate progression-free survival (PFS) and objective response rate (ORR) to avoid detection bias of local investigators. However, it is not clear whether the bias has been adequately identified, or to what extent it consistently distorts the results. Therefore, the objective of this study was to evaluate the detection bias in oncological open-label trials by confirming whether local investigators overestimate the PFS and ORR compared with the findings of central reviewers.

**Design:**

Meta-epidemiological study.

**Data sources:**

MEDLINE via PubMed from 1 January 2010 to 30 June 2021.

**Eligibility criteria for selecting studies:**

Open-label, parallel-group superiority, randomised trials of anticancer drugs that adjudicated PFS or ORR by both central reviewers and local investigators.

**Review methods:**

We assessed the values for the same outcome (PFS and ORR) adjudicated by both central reviewers and local investigators. A random-effects model was used to estimate the ratio of HR (RHR) for PFS and the ratio of OR (ROR) for ORR between central reviewers and local investigators. An RHR lower than 1 and an ROR higher than 1 indicated an overestimation of the effect estimated by local investigators.

**Results:**

We retrieved 1197 records of oncological open-label trials after full-text screening. We identified 171 records (PFS: 149 records, ORR: 136 records) in which both central reviewers and local investigators were used, and included 114 records (PFS: 92 records, ORR: 74 records) for meta-analyses. While the RHR for PFS was 0.95 (95% CI 0.91 to 0.98), the ROR of ORR was 1.00 (95% CI 0.91 to 1.09). The results remained unchanged in the prespecified sensitivity analysis.

**Conclusions:**

This meta-epidemiological study found that overestimation of local investigators has a small impact on evaluating PFS and ORR in oncological open-label trials. However, a limitation of this study is that it did not include data from all trials; hence, the results may not fully evaluate detection bias. The necessity of central reviewers in oncological open-label trials needs to be assessed by further studies that overcome this limitation.

**Trial registration number:**

CTR-UMIN000044623.

**What is already known on this topic**

- The US Food and Drug Administration and European Medicines Agency recommend the use of central reviewers in oncological open-label trials to avoid detection bias; however, this recommendation is not based on evidence. Previous meta-epidemiological studies have compared progression-free survival (PFS) or objective response rate (ORR) adjudicated by central reviewers and local investigators, but have not clearly identified an overestimation by local investigators. However, these studies did not specifically focus on open-label trials, and there is a concern that an adequate number of studies were not included through an appropriate search strategy.

**What this study adds**

- This meta-epidemiological study found that only a small fraction of the studies on anticancer drugs adjudicated PFS or ORR using both central and local investigators, and only half of these studies reported both outcomes. A meta-analysis of these studies showed that PFS may have been slightly overestimated by local investigators, while ORR was not. A sensitivity analysis which used a range of assumptions did not change these results. Our study suggests that the impact of the differences between central and local adjudications is not substantial. However, as this study did not access data from all trials, the results may not fully evaluate detection bias. Among the studies that claimed both assessors were used, half of them only reported the results from one assessor, indicating potential selective outcome reporting bias. Until the findings of this study

**are validated by studies that overcome this limitation, it is desirable to establish central reviewers in oncological open-label trials.**

**How might this affect research, practice or policy**

- It is expected that the results of this study will lead to a trend towards reporting outcomes adjudicated by both central and local investigators. Additional meta-epidemiological studies would then be conducted to validate this study and accumulate knowledge on detection bias in open-label trials of anticancer drug.

## Introduction

Detection bias can systematically distort the results of randomised controlled trials.^1^ This bias, also known as observer bias or measurement bias, can occur when the outcomes are subjective and the adjudicators are not blinded (or masked), leading them to evaluate the results optimistically. In general medicine, several meta-epidemiological studies have assessed the magnitude of detection bias by comparing the same outcomes between blinded and non-blinded adjudicators, with conflicting findings and a lack of consensus.^2–5^ Recently, the MetaBLIND study found no difference in the estimated treatment effect between trials with and without blinded outcome adjudicators; however, since they compared the outcomes in different trials, the study had a risk of confounding.^6^ Furthermore, these meta-epidemiological studies have diverse specialties and outcomes, and whether they can be generalised to specific outcomes in oncology requires validation.

Open-label trials are more common in oncological clinical trials than in non-oncological trials,^7^ and there has been a trend towards using progression-free survival (PFS) and objective response rate (ORR) as primary outcomes in oncological trials rather than overall survival (OS).^8^ This is due to the abbreviated time required to evaluate efficacy, smaller sample size requirements and lack of subsequent treatments. However, PFS and ORR involve more subjective judgement than OS, particularly in open-label settings. Given recent trends, detection bias is a particular concern in oncological clinical trials.

The US Food and Drug Administration recommends the use of central reviewers blinded to study treatment to verify tumour assessment to minimise bias in oncological clinical trials when the primary endpoint is PFS or ORR.^9^ The European Medicines Agency also emphasises the importance of blinded independent central reviewers, especially when the majority of events are based on imaging rather than clinical progression.^10^ However, previous oncological meta-epidemiological studies have yielded inconsistent findings and do not necessarily demonstrate overestimation by local investigators.^11–15^ Nevertheless, these studies did not focus on open-label trials, and there is a concern that an adequate number of studies were not included through an appropriate search strategy. Therefore, while the importance of central reviewers is widely recognised in oncology, there is limited evidence to support their importance.

This meta-epidemiological study focused on open-label trials of anticancer drugs and aimed to evaluate detection bias by confirming whether local investigators overestimate the PFS and ORR compared with the estimates by central reviewers.

## Methods

### Study design

This was a meta-epidemiological study of randomised controlled trials registered in the UMIN Clinical Trial Registry (CTR-UMIN000044623). The protocol was published as a preprint in the Open Science Foundation^16^ and descriptive summary results of the identified studies have been reported elsewhere.^17^ The study was conducted in accordance with the Preferred Reporting Items for Systematic Reviews and Meta-Analyses (PRISMA) statement.^18^

### Eligibility criteria

#### Types of studies

We included open-label, parallel-group superiority and randomised trials that investigated the efficacy of anticancer drugs. Non-inferiority and equivalence trials were excluded because the null hypothesis was different from that of the superiority trials. We also excluded records that were not in English language.^19^

#### Types of participants

We focused on solid tumours and excluded haematological diseases, including leukaemia, lymphoma and multiple myeloma. Because haematological cancers are evaluated biologically, their evaluations are less likely to be influenced by adjudicators than solid tumours. Therefore, we only included solid cancers of all histological types and stages.

#### Types of interventions

Eligible interventions included molecularly targeted therapy, immune checkpoint inhibitors, immune therapy, chemotherapy or hormone therapy; and eligible comparisons included standard therapy, supportive care or no treatment. Combination therapies were also included (eg, therapy A plus standard therapy vs standard therapy). Owing to heterogeneity, we excluded neoadjuvant and adjuvant interventions.

#### Types of outcomes

We included trials that used PFS or ORR as measurements of treatment efficacy.

### Information sources

We identified relevant trials from the MEDLINE database.

### Search strategy

We searched MEDLINE from 1 January 2010 to 30 June 2021. Online supplemental appendix lists the search terms used on 1 July 2021. Furthermore, we conducted a manual search of the reference lists attached to relevant articles. We restricted the search period to 2010 to allow the possibility of contacting the corresponding authors.

10.1136/bmjebm-2023-112332.supp1 Supplementary data

### Selection process

**Figure 1:**
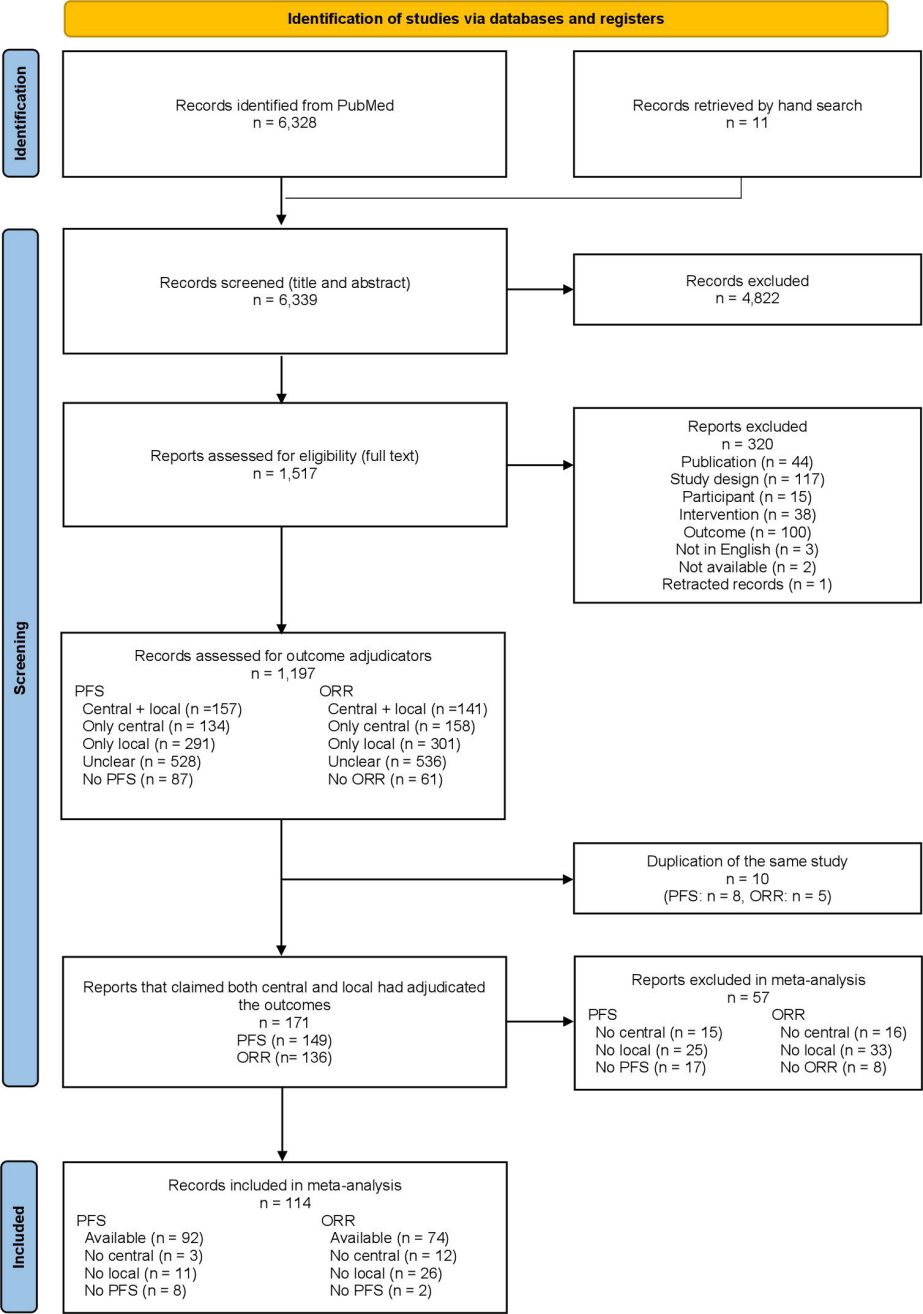
Flow chart of the study selection. ORR, objective response rate; PFS, progression-free survival.

First, seven independent pairs of researchers (SF and TY; SF and YF; SF and SY; SF and NN; YL and YK; TY and MK; TY and MT) screened the titles and abstracts of the records identified by the literature search. Second, different seven pairs of researchers (SF and YL; SF and YK; SF and TY; SF and YF; SF and SY; SF and MK; SF and MT) independently screened the full texts of the records. After full-text screening, we classified the articles by outcome adjudicators into four categories: ‘central and local’, ‘only central’, ‘only local’ and ‘unclear’. Based on this classification, we selected articles in which the outcome was adjudicated by both the central and local investigators. Any disagreement was resolved through discussion or consultation with another pair or TAF if necessary. The PRISMA flow chart (figure 1) summarises the reasons for excluding studies.

### Data collection process

Eight pairs of researchers independently collected detailed information from each trial using a prepiloted form (SF and YL; SF and YK; SF and TY; SF and YF; SF and SY; SF and MK; SF and MT; SF and NN). When no details were reported for the data items, the corresponding authors were queried.

### Data items

We collected the data items as follows:

1. General information of the study: publication journal, publication year, authors, trial registry number (National Clinical Trial number, European Union Clinical Trial number and others), trial phase (phase II, phase III and others), cancer type and number of randomised participants.
2. Intervention and comparison information: drug name, drug classification (targeted therapy, immune checkpoint inhibitor, immune therapy, chemotherapy or hormone therapy), comparison treatment (standard treatment, BSC or no treatment) and treatment line.
3. Outcome information: primary outcome, response criteria (response evaluation criteria in solid tumours (RECIST) 1.0,^20^ RECIST V.1.1,^21^ WHO criteria^22^ and others), point estimates and 95% CIs of the HR of PFS assessed by central reviewers (HR_central_), HR of PFS assessed by local investigators (HR_local_), OR of ORR assessed by central reviewers (OR_central_) and OR of ORR assessed by local investigators (OR_local_). If the study did not report the 95% CIs, we collected the p value or the other CI (eg, the 90% CIs) to obtain the SE.^23^

### Statistical analysis

The characteristics of the included studies were summarised as numbers and relative frequencies for categorical variables. Our primary outcomes were the ratio of HR (RHR) and the ratio of OR (ROR) between HR_central_ (or OR_central_) and HR_local_ (or OR_local_). We performed a meta-analysis of RHR using the two-step process proposed by Sterne *et al*.^24^ Within each trial, we divided HR_local_ by HR_central_ to calculate the RHR and divided OR_local_ by OR_central_ to calculate the ROR. An RHR<1 indicates that the local investigators overestimate the effect compared with the findings of central reviewers, which suggests the existence of a detection bias. In order to calculate the SE of RHR in each trial, we transformed RHR to log RHR and calculated the SE of the logRHR (SE(logRHR)) using the following equation:

SE(logRHR)=Var(logHRcentral)+Var(logHRlocal)−2×ρ×SE(logHRcentral)×SE(logHRlocal)

where ρ is correlation coefficients between HR_central_ and HR_local_ in each trial. Then, we performed a random-effects meta-analysis using the inverse variance method. We were unable to calculate ρ without individual patient data. We, therefore, assumed no dependency between HR_central_ and HR_local_ in each trial (ρ=0) for the main analysis, as it is the most conservative approach (ie, it generates the largest SE estimation and hence widest CIs).^4^ To test robustness, we also performed sensitivity analyses assuming ρ=0.25, 0.50, 0.75 and 0.95. We presented a forest plot to visualise the RHR in all included studies. We evaluated the heterogeneity of RHR across different trials using the tau^2^ and I^2^ statistics. We prespecified and explored whether the direction or magnitude of detection bias would vary in the following subgroups: (1) trial phases, (2) cancer types and (3) drug classifications. We calculated the p values for interactions using a meta-regression model. We performed a meta-analysis of the ROR in the same manner. Note that unlike RHR, an ROR>1 indicates that the local investigators overestimate the effect compared with the findings of central reviewers, indicating detection bias. The ‘metafor’ package (V.3.8–1)^25^ in R (V.4.0.3; R Foundation for Statistical Computing, Vienna, Austria) was used for meta-analysis. The collected data and codes used for our analysis are provided in the online supplemental materials.

### Patient and public involvement

Patients and members of the public were not involved in this study because it was conducted to answer a methodological question that was not directly dependent on patient priorities, experiences or participant preferences.

## Results

### Study selection

Figure 1 illustrates the study selection process. We identified 6339 records, 1517 of which were eligible after screening titles and abstracts. From 1517 records, we retrieved 1197 eligible records after full-text screening and assessed the adjudicators of PFS and ORR. A total of 181 records (PFS: 157 records, ORR: 141 records) were adjudicated by both central reviewers and local investigators (online supplemental table 1), and the trend of the prevalence of outcome adjudicators did not change from 2010 to 2021 in both PFS (online supplemental figure 2) and ORR (online supplemental figure 3). Of the 181 records, we excluded 10 due to data duplication and included the remaining records that could extract the outcomes adjudicated by both central reviewers and local investigators. Finally, we included 114 records in this analysis, of which 92 were analysed for PFS and 74 for ORR. In other words, among the records that we judged were adjudicated by both central reviewers and local investigators for PFS, only 61.7% (92/149) reported results from both assessors and 54.4% (74/136) for ORR. Online supplemental materials list the exclusion criteria for records during the full-text screening stage (n=320), data extraction stage (n=57) and duplication records stage (n=10). We sent 121 emails to the corresponding authors to request details of the data (26 October 2022) and received 15 responses, of which only one provided sufficient information. The other 14 corresponding authors stated that they could not access the data.

### Study characteristics

Table 1 presents the characteristics of the included records. The analysis included 114 records, 92 for PFS and 74 for ORR. The majority of the trials were phase III (n=74); the most common tumour types were non-small cell lung cancer (n=29), breast cancer (n=22) and renal cell cancer (n=14); and the most common type of drug was targeted therapy (n=70). PFS was the primary outcome in 82 records, whereas ORR was the primary outcome in 13. Most of the reports were from high-impact factor (>10) journals (n=102), with only 5 funded by the public sector.

**Table 1:** Characteristics of included studies (n=114)

	No (%)
Reported outcome	
PFS and ORR	52 (45.6)
Only PFS	40 (35.1)
Only ORR	22 (19.3)
Trial phase	
Phase III	74 (64.9)
Phase II	40 (35.1)
Cancer type	
Non-small cell lung cancer	29 (25.4)
Breast cancer	22 (19.3)
Renal cell cancer	14 (12.3)
Melanoma	9 (7.9)
Ovarian cancer	8 (7.0)
Head and neck cancer	5 (4.4)
Gastric and gastro-oesophageal junction adenocarcinoma	4 (3.5)
Sarcoma	4 (3.5)
Colorectal cancer	3 (2.6)
Gastrointestinal stromal tumour	3 (2.6)
Hepatocellular cancer	2 (1.8)
Pancreatic cancer	2 (1.8)
Others*	10 (8.8)
Drug classification of intervention	
Targeted therapy	70 (61.4)
Chemotherapy	26 (22.8)
Immune checkpoint inhibitor	15 (13.2)
Immunotherapy	3 (2.6)
Treatment line	
≥1st line	59 (51.8)
≥2nd line	47 (41.2)
≥3rd line	8 (7.0)
Primary outcome in the published paper	
PFS	82 (71.9)
ORR	13 (11.4)
OS	6 (5.3)
OS and PFS	7 (6.1)
Others†	6 (5.3)
Blinding of central reviewers	
Blinding	72 (63.2)
Unclear	42 (36.8)
Impact factor (2021)	
≥10	102 (89.5)
<10	12 (10.5)
Funding	
Industry	106 (93.0)
Public	5 (4.4)
Unclear	3 (2.6)

*Endometrial carcinoma, glioblastoma, liposarcoma or leiomyosarcoma, malignant mesothelioma, neuroendocrine tumours, pleural mesothelioma, prostate cancer, small cell lung cancer, squamous cell carcinoma of the lung.

†OS and ORR, OS and ORR and PFS, time-to-treatment failure, tumour shrinkage, safety, incidence of cutaneous squamous cell carcinoma, PFS, response rate, duration of response and safety.

ORR, objective response rate; OS, overall survival; PFS, progression-free survival.

### Primary outcome: RHR and ROR

**Figure 2:**
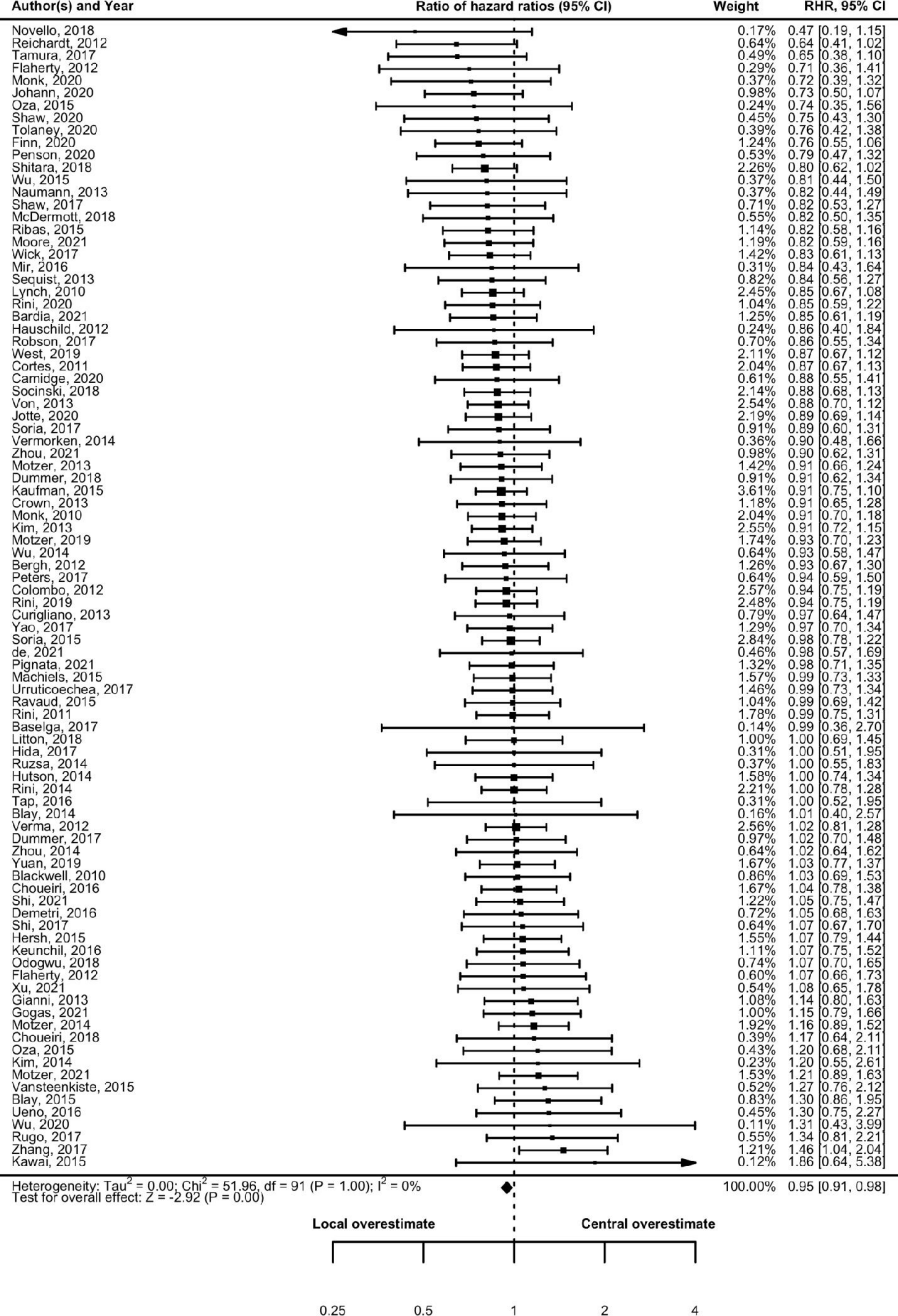
Comparison of treatment effect estimates (HR) between central reviewers and local investigators. RHR, ratio of HR.

**Figure 3:**
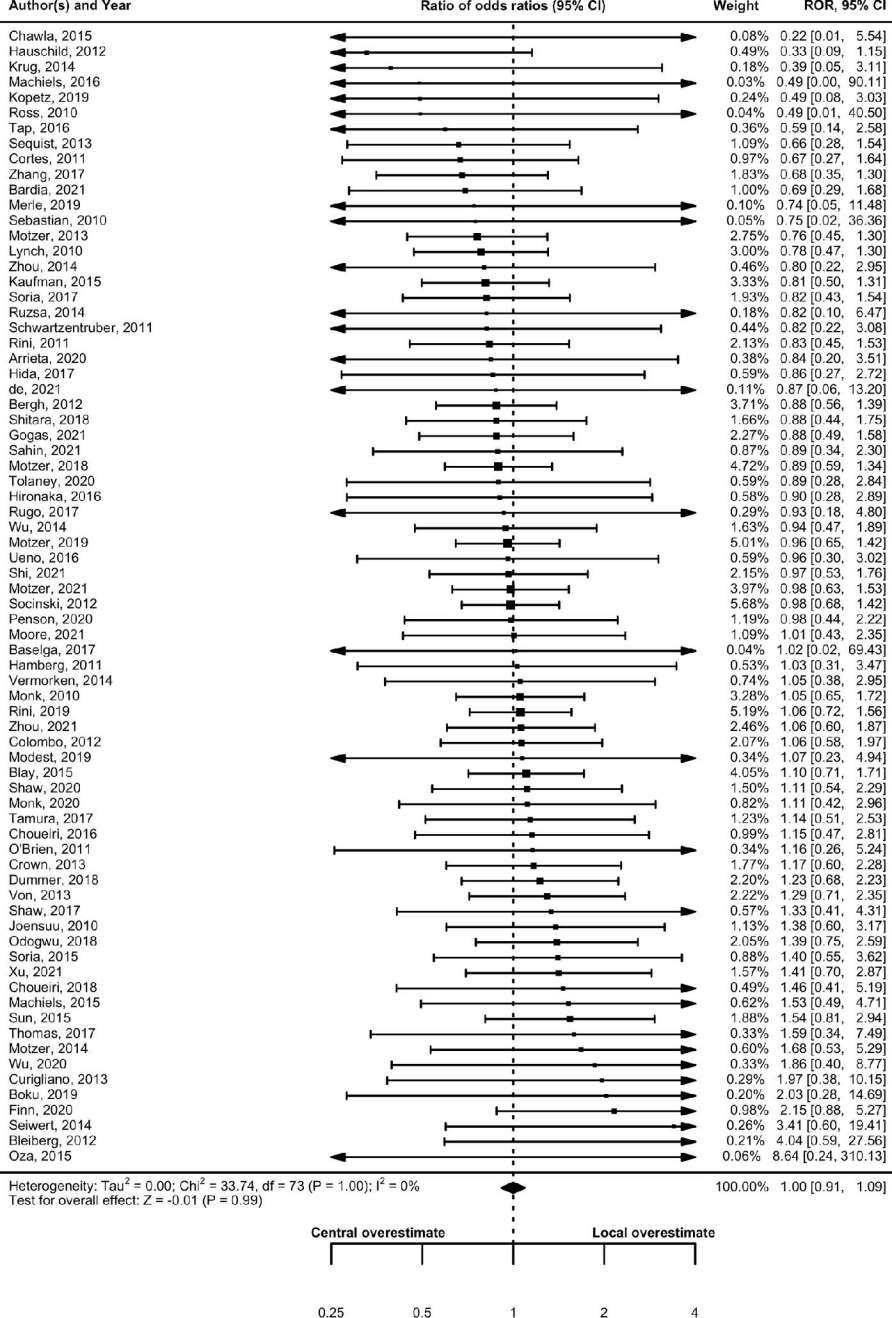
Comparison of treatment effect estimates (OR) between central reviewers and local investigators. ROR, ratio of OR.

Table 2 and figure 2 present the RHR for PFS. Under the assumption of no dependency between central reviewers and local investigators in each trial (ρ=0), the RHR for PFS was 0.95 (95% CI 0.91 to 0.98), indicating that local investigators slightly overestimated the HR compared with the findings of central investigators. No heterogeneity was detected in the meta-analysis (tau^2^=0; I^2^=0%; p>0.99). Table 3 and figure 3 present ROR for ORR and, in contrast, the ROR for ORR was 1.00 (95% CI 0.91 to 1.09), and no heterogeneity was observed in meta-analyses (tau^2^=0; I^2^=0%; p>0.99). Sensitivity analysis showed the RHR for PFS and ROR for ORR under the assumption of dependency between the central reviewers and local investigators in each trial (online supplemental table 2 and online supplemental figures 4a–4d and 5a–5d). The RHR remained constant at 0.95, and the upper 95% CIs did not exceed 1.00, indicating that local investigators consistently overestimated the HR for PFS compared with the findings of central reviewers under any assumptions of dependency. Conversely, the ROR ranged from 1.00 to 1.03, with lower 95% CIs consistently below 1.00, indicating that local investigators did not overestimate the OR for ORR under any assumption of dependency. Heterogeneity increased for both PFS and ORR from ρ=0 (independent) to ρ=0.95 (nearly completely dependent). Tables 2 and 3 and online supplemental figures 6a,b and 8a,b show the prespecified subgroup analyses of trial phases, cancer types and drug classifications. No interactions were observed between these subgroups for either the PFS or ORR.

**Table 2:** Estimated ratios of HRs between central and local adjudications in all the studies and in subgroups

	No of records	RHR (95% CI)	Tau^2^	I^2^	P value for heterogeneity	P value for interaction
Overall	92	0.95 (0.91 to 0.98)	0.00	0%	>0.99	
Trial phase						0.58
Phase III	68	0.94 (0.91 to 0.98)	0.00	0%	>0.99	
Phase II	24	0.97 (0.87 to 1.09)	0.00	0%	0.96	
Cancer type						0.86
Non-small cell lung cancer	23	0.91 (0.85 to 0.99)	0.00	0%	>0.99	
Breast cancer	20	0.98 (0.90 to 1.05)	0.00	0%	0.80	
Renal cell cancer	13	1.00 (0.92 to 1.09)	0.00	0%	0.95	
Melanoma	8	0.98 (0.85 to 1.13)	0.00	0%	0.86	
Ovarian cancer	8	0.91 (0.80 to 1.03)	0.00	0%	0.93	
Gastrointestinal stromal tumours	3	0.91 (0.58 to 1.42)	0.09	59%	0.08	
Head and neck cancer	3	0.97 (0.76 to 1.24)	0.00	0%	0.96	
Sarcoma	3	1.14 (0.70 to 1.85)	0.00	0%	0.60	
Pancreatic cancer	2	0.88 (0.70 to 1.12)	0.03	37.7%	0.21	
Endometrial carcinoma	1	0.74 (0.35 to 1.56)				
Gastric and gastro-oesophageal junction cancer	1	0.80 (0.62 to 1.02)				
Glioblastoma	1	0.83 (0.61 to 1.13)				
Hepatocellular cancer	1	0.76 (0.55 to 1.06)				
Liposarcoma or Leiomyosarcoma	1	1.05 (0.68 to 1.63)				
Malignant mesothelioma	1	0.98 (0.57 to 1.69)				
Neuroendocrine tumours	1	0.97 (0.70 to 1.34)				
Prostate cancer	1	0.73 (0.50 to 1.07)				
Squamous cell carcinoma of the lung	1	0.98 (0.78 to 1.22)				
Drug classification						0.45
Targeted therapy	62	0.95 (0.91 to 1.00)	0.00	0%	>0.99	
Immune checkpoint inhibitor	12	0.90 (0.83 to 0.98)	0.00	0%	0.73	
Chemotherapy	18	0.97 (0.89 to 1.05)	0.00	0%	0.71	

RHR, ratio of the HR.

**Table 3:** Estimated ratios of ORs between central and local adjudications in all the studies and in subgroups

	No of records	ROR (95% CI)	Tau^2^	I^2^	P value for heterogeneity	P value for interaction
Overall	74	1.00 (0.91 to 1.09)	0.00	0%	>0.99	
Trial phase						0.30
Phase III	45	0.98 (0.89 to 1.08)	0.00	0%	>0.99	
Phase II	29	1.14 (0.88 to 1.47)	0.00	0%	>0.99	
Cancer type						0.92
Non-small cell lung cancer	18	1.01 (0.85 to 1.20)	0.00	0%	0.99	
Breast cancer	14	0.94 (0.76 to 1.15)	0.00	0%	0.95	
Renal cell cancer	9	0.96 (0.81 to 1.14)	0.00	0%	0.95	
Head and neck cancer	5	1.36 (0.71 to 2.61)	0.00	0%	0.79	
Ovarian cancer	5	1.05 (0.77 to 1.42)	0.00	0%	>0.99	
Gastric and gastro-oesophageal junction cancer	4	0.93 (0.57 to 1.52)	0.00	0%	0.89	
Melanoma	4	0.91 (0.62 to 1.34)	0.00	0%	0.47	
Colorectal cancer	3	1.22 (0.42 to 3.50)	0.09	9.9%	0.29	
Hepatocellular cancer	2	1.94 (0.83 to 4.55)	0.00	0%	0.79	
Pancreatic cancer	2	1.22 (0.72 to 2.06)	0.00	0%	0.95	
Sarcoma	2	0.50 (0.13 to 1.91)	0.00	0%	0.59	
Endometrial carcinoma	1	8.64 (0.24 to 310.13)				
Gastrointestinal stromal tumours	1	1.10 (0.71 to 1.71)				
Malignant mesothelioma	1	0.87 (0.06 to 13.20)				
Pleural mesothelioma	1	0.39 (0.05 to 3.11)				
Small cell lung cancer	1	1.16 (0.26 to 5.24)				
Squamous cell carcinoma of the lung	1	1.40 (0.55 to 3.62)				
Drug classification						0.99
Targeted therapy	41	1.00 (0.87 to 1.14)	0.00	0%	>0.99	
Immune checkpoint inhibitor	10	1.00 (0.84 to 1.18)	0.00	0%	0.90	
Immunotherapy	3	0.89 (0.36 to 2.21)	0.00	0%	0.56	
Chemotherapy	20	1.01 (0.86 to 1.19)	0.00	0%	0.97	

ROR, ratios of OR.

## Discussion

### Principal findings

This meta-epidemiological study found that local investigators tended to slightly overestimate the HR for PFS compared with the findings of central reviewers in oncological open-label trials. In contrast, there was no evidence of overestimation in ORR. These results remained consistent in the sensitivity analysis which accounted for various assumptions, and the subgroup analysis did not identify any factors that might influence the findings. However, it is important to note that these results were based on a small subset of oncological open-label trials, and only approximately half of the reports that claimed the outcomes were adjudicated by both central reviewers and local investigators reported both results.

### Strengths and limitations of the study

This study has several strengths. First, because it excluded any terms related to outcome adjudicators in its search strategy, this study represents the most comprehensive meta-analysis to date, including the largest number of studies of any existing reports. Second, we were able to provide a breakdown of adjudicated outcomes in recent oncological open-label trials. This enabled us to detect how many studies adjudicated PFS and ORR by central reviewers and local investigators, as well as the corresponding number of reported outcomes. Third, the results remained unchanged after sensitivity analysis, highlighting their robustness. Fourth, as our study compared the same outcomes between central and local investigators within the same trial, the risk of confounding was low.

Although this study has several strengths, it also has some limitations. First, the records in which both central reviewers and local investigators adjudicated the outcomes were only a small proportion of the total number of oncological open-label trials. Furthermore, only half of all reports were available for analysis, with the rest not reporting results from either assessor. Additionally, our preliminary study showed that outcome adjudicators might differ from those prespecified in the protocol or trial registry,^17^ suggesting the possibility of selective outcome reporting bias in our results. In other words, among studies that claimed that both central reviewers and local investigators adjudicated the outcome, we expect studies that reported results by both assessors (ie, studies included in our analysis) to show less significant differences between the two assessors. Moreover, when we contacted the investigators of those trials that reported only central or local adjudicators’ outcomes, very few investigators provided data regarding local adjudicator outcomes. Because trial sponsors are unlikely to publish the results of trials in which the central and local adjudications are inconsistent, the low response rate may underestimate the extent of detection bias by local investigators due to selective outcome reporting bias. However, it should be noted that although the number of studies included in the analysis was limited, they showed a slight overestimation in the HR of PFS by local investigators. Actual detection bias could be greater in real-world settings. Second, we assumed that the dependence (ρ) between central and local adjudicators in all studies was 0 in the primary analysis, and between 0.25 and 0.95 in the sensitivity analysis. However, the presence of no dependence (ρ=0) between central and local adjudications is implausible, and the dependence may vary from trial to trial; therefore, this assumption may not be entirely accurate. A more rigorous synthesis could be achieved by calculating the true dependence in each study using individual patient data. Nevertheless, as our sensitivity analysis indicated that changing the values of ρ from 0 to 0.95 did not affect the final results, we assume that the variation in ρ observed among the studies would not affect the final RHR or ROR. Third, the period of the search was limited to studies published after 2010. Although this meta-analysis includes more studies than previously published reviews, this search strategy was not entirely systematic.

### Comparison with other studies

Regarding general medicine, the MetaBLIND study, which evaluated the effect of blinding outcome assessors to the intervention, did not find an apparent overestimation by non-blinded assessments,^6^ However, as this meta-epidemiological study compared the outcomes of different trials, there was a risk of confounding. Contrarily, when examining studies that compared blinded and non-blinded outcome adjudicators within the same trials, several previous meta-epidemiological studies found an overestimation of non-blinded adjudication by 27%–36%.^2–4^ Nevertheless, as these reviews did not focus on the same medical field and outcomes and included both double-blinded and open-label trials, these findings may not be generalisable to oncology.

Narrowing the focus to oncology and the outcomes to PFS and ORR, several meta-epidemiological studies have been published to date, with inconsistent findings. Two studies that evaluated PFS found no apparent differences between central and local investigators.^12 13^ However, these studies did not focus exclusively on open-label trials, and included 36 and 21 open-label trials, respectively. Of the three studies that evaluated ORR, one study found an overestimation by local investigators of 17.5% in 33 trials. However, this was limited to phase II trials and did not distinguish between double-blind and open-label trials.^14^ Two studies, which included 22 and 23 open-label trials, found no evidence of overestimation.^11 12^ The most recent published review included 38 and 33 open-label trials in the meta-analysis of PFS and ORR, respectively, and found that local investigators overestimated PFS but not ORR, which is consistent with the findings of our study.^15^ In comparison to these meta-epidemiological studies, we specifically focused on open-label trials and included a large number of reports using the updated search strategy. Our results show that local investigators slightly overestimated PFS by 5%, but not did not overestimate ORR. While there were some discrepancies in the results between the studies; however, the impact of the discrepancies between central and local adjudications is not substantial.

### Mechanisms and implications

There are many possible reasons for discrepancies between central reviewers and local investigators in the adjudication of tumour outcomes.^26^ Local investigators often lack formal training in radiology, and adjudication may be influenced by knowledge of a patient’s clinical status. Central reviewers help ensure consistency in data collection and adjudication across sites, reducing the potential for measurement error and bias. In this study, we found detection bias in the estimation of the HR of PFS but not in the estimation of the OR of ORR. This could be because PFS is a more subjective outcome than ORR. ORR may be less prone to bias, as it is usually defined according to imaging evaluation using manuals such as RECIST, making it more objective. Although there was bias in HR of PFS, the bias was not marked and may not have significantly impacted the estimation and interpretation of the effect. This suggests that central reviewers are not necessary in oncological open-label trials.

However, it is important to note that methodological biases may have distorted the true values and affected these results. In other words, the results represent three possibilities: (1) low risk of detection bias, (2) underestimation of detection bias due to selective outcome reporting bias and (3) underestimation of detection bias due to the use of central reviewers. (1) If there is no methodological bias in this study, these results are true values and there is low risk of detection bias in oncological open-label trials. (2) If selective outcome reporting is present in this study (ie, the analysed studies that reported results from both assessors tended to show smaller differences between the two reviewers than studies that reported results from only one assessor), these results might underestimate the detection bias. (3) If the central adjudicators’ judgement was influenced by the local investigators,^27^ or the influence of informative censoring is higher than that assumed,^28^ the difference between the central and local adjudications would be reduced, resulting in an underestimation of the detection bias. Informative censoring occurs when patients whose progression is judged by local investigators but not by central reviewers are treated as censored. Previous studies, including this study, have only analysed openly available data, so it is difficult to verify these possibilities. To address this challenge, all studies with central adjudication should also report the results of the local investigators, and a meta-analysis of the ratios should be performed. We expect that further epidemiological studies will accumulate over time, enabling our findings to be investigated.

## Conclusions

This meta-epidemiological study found that compared with the findings of central reviewers, local investigators may slightly overestimate the PFS, but not the ORR, in oncological open-label oncological trials. These findings suggest that detection bias of local investigators may not be substantial in the field of oncology. However, this analysis did not extract data from all identified trials and thus may not reflect true detection bias in oncological trials. Further studies that overcome this limitation are necessary before conclusions can be drawn.

## Data Availability

Data are available in a public, open access repository. The collected data and codes used for our analysis are provided in online supplemental materials (https://github.com/SatoshiFunada/2023_detection_bias).
