# Supplementary material for: Detection bias in open-label trials of anticancer drugs: a meta-epidemiological study
Source: BMJ Evid Based Med. 2023 Aug 16;28(6):372–82. doi: 10.1136/bmjebm-2023-112332 (PMC10715523; doi:10.1136/bmjebm-2023-112332)
Supplement: Supplementary data [file bmjebm-2023-112332supp001.pdf]

Supplementary Appendix

We used the search terms to search the MEDLINE via PubMed (1 Jan 2010 to 30 June 2021) as follows:

#1 "neoplasms"[MeSH Terms] OR "cancer*"[Title/Abstract] OR "carcinoma*"[Title/Abstract] OR "oncol*"[Title/Abstract] OR "tumor*"[Title/Abstract] OR "tumour*"[Title/Abstract] OR "adenocarcinoma*"[Title/Abstract] NOT Leukemia[MeSH Terms] NOT Lymphoma[MeSH Terms] NOT “Multiple Myeloma” [MeSH Terms]

#2 "random*"[Title/Abstract]

#3 "Antineoplastic Agents"[MeSH Terms] OR "immunotherapy"[All Fields] OR "Immune Checkpoint Inhibitors"[All Fields] OR "target therapy"[All Fields] OR "hormone"[All Fields] OR "chemotherapy"[All Fields]

#4 "humans"[MeSH Terms]

#5"english"[Language]

#6 randomizedcontrolledtrial[Filter]

#7 2010/01/01:2021/06/30[Date - Publication]

#8 adjuvant[Title] OR neoadjuvant[Title]

#9 "placebo"[Title] OR "blind"[Title]

#1 AND #2 AND #3 AND #4 AND #5 AND #6 AND #7 NOT #8 NOT #9

Funada S, *et al. BMJ EBM* 2023; 28:373–383. doi: 10.1136/bmjebm-2023-112332

Supplementary Figure 2. The trend in PFS adjudicators from 2010 to 2021

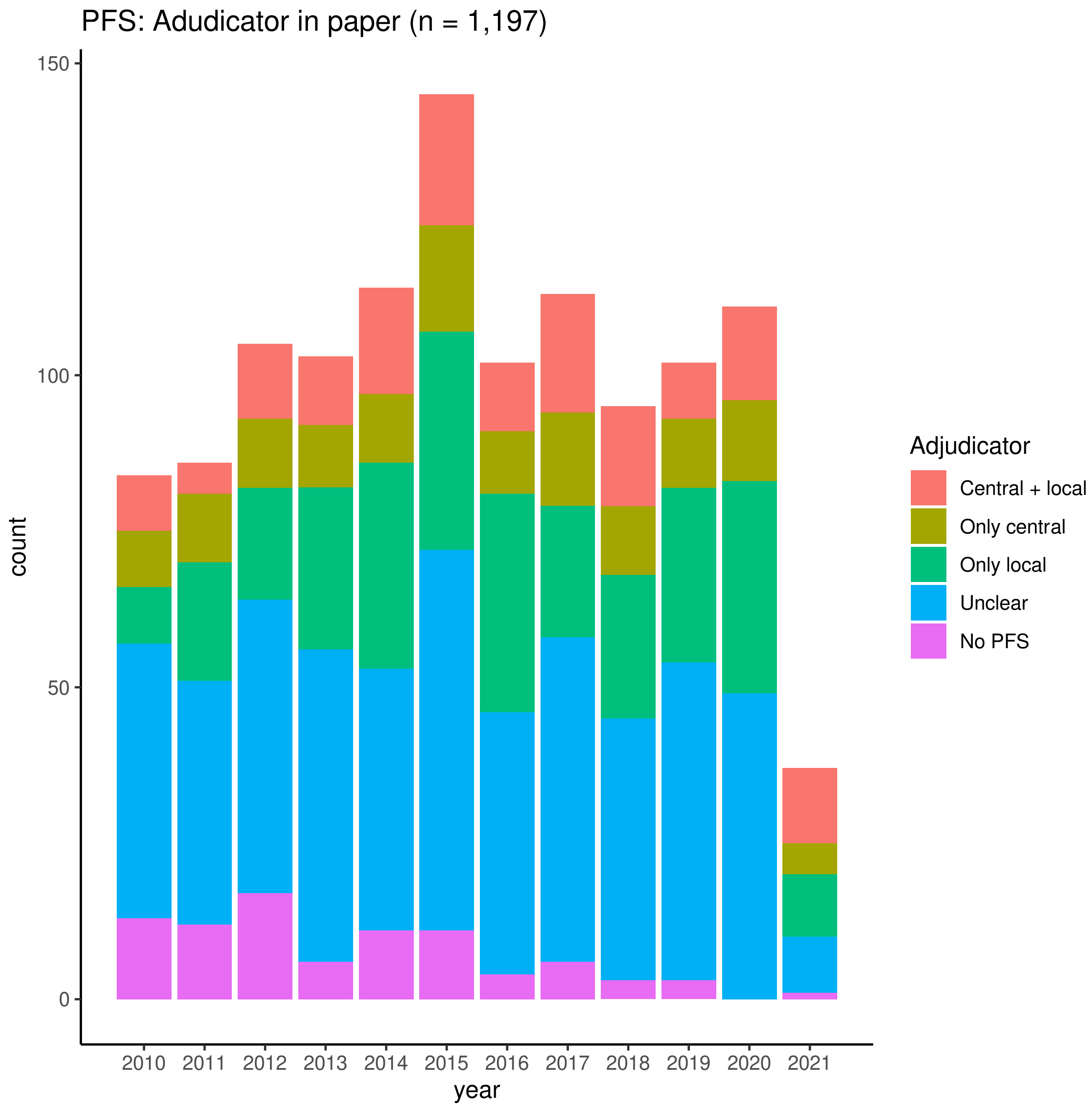

Funada S, *et al. BMJ EBM* 2023; 28:373–383. doi: 10.1136/bmjebm-2023-112332

Supplementary Figure 3. The trend in ORR adjudicators from 2010 to 2021

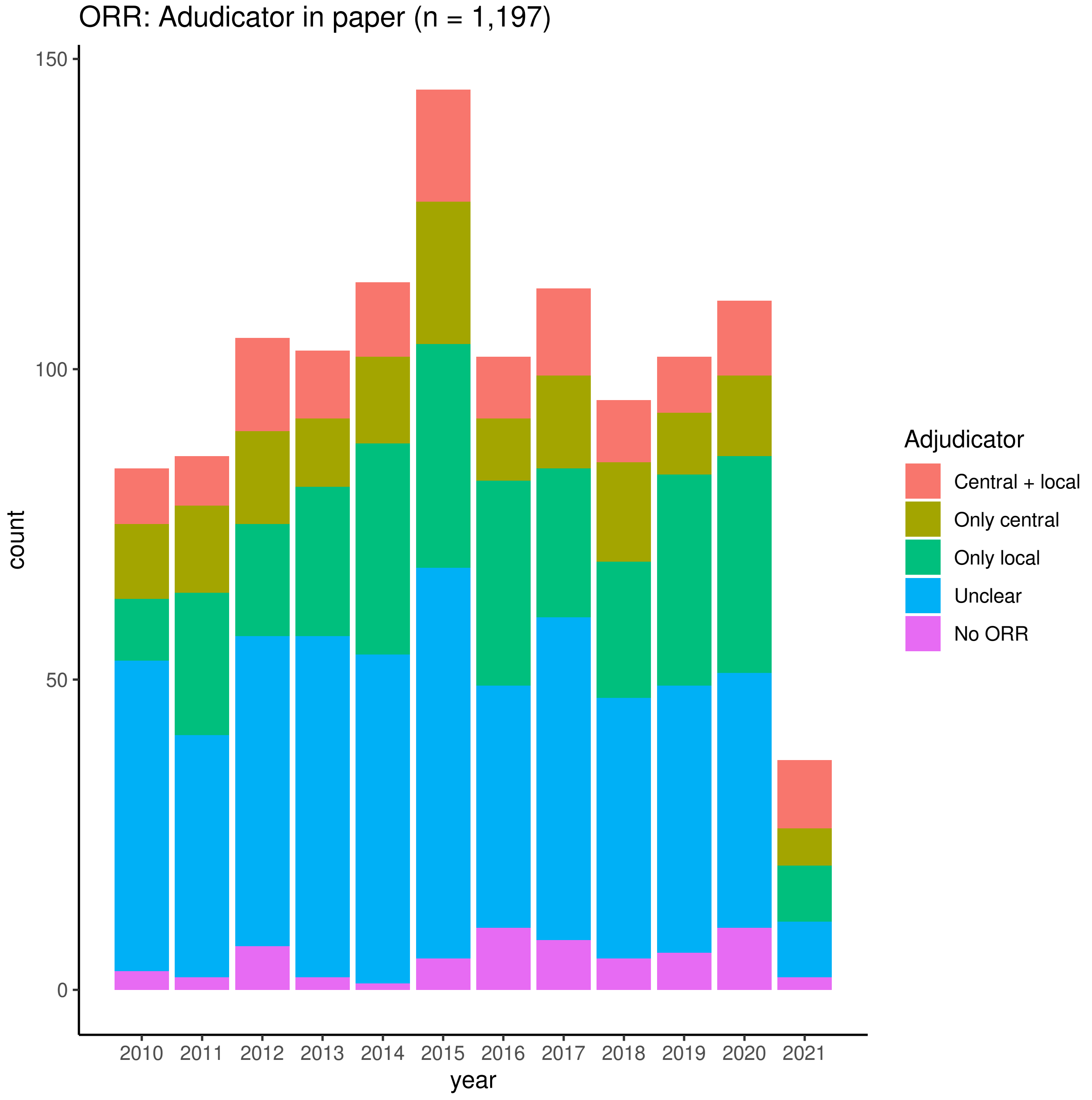

Funada S, *et al. BMJ EBM* 2023; 28:373–383. doi: 10.1136/bmjebm-2023-112332

Supplementary Figure 4a. Comparison of treatment effect estimates (Hazard Ratio) between central reviewers and local investigators. Sensitivity analysis (ρ = 0.25).

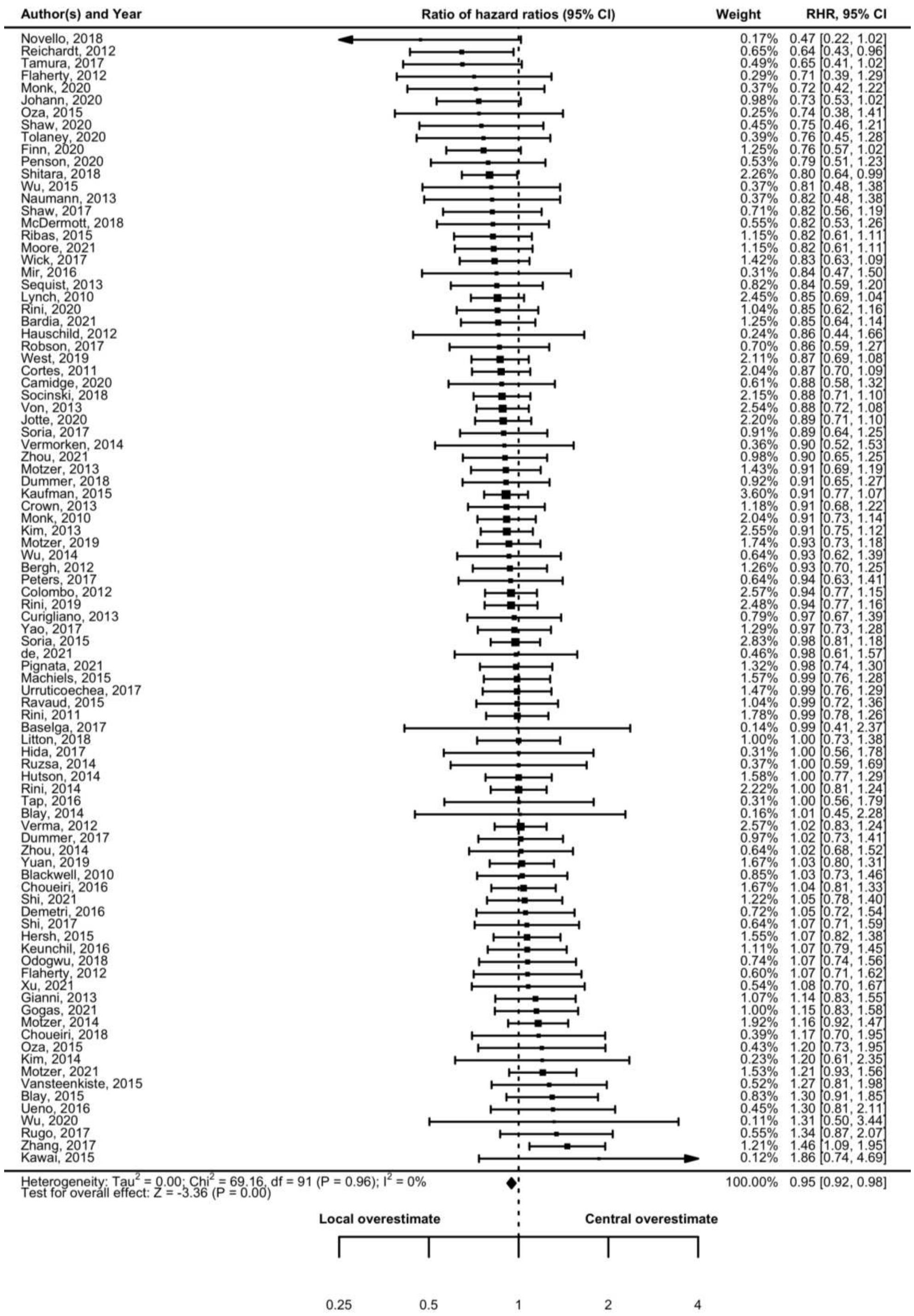

Funada S, *et al. BMJ EBM* 2023; 28:373–383. doi: 10.1136/bmjebm-2023-112332

Supplementary Figure 4b. Comparison of treatment effect estimates (Hazard Ratio) between central reviewers and local investigators. Sensitivity analysis (ρ = 0.50).

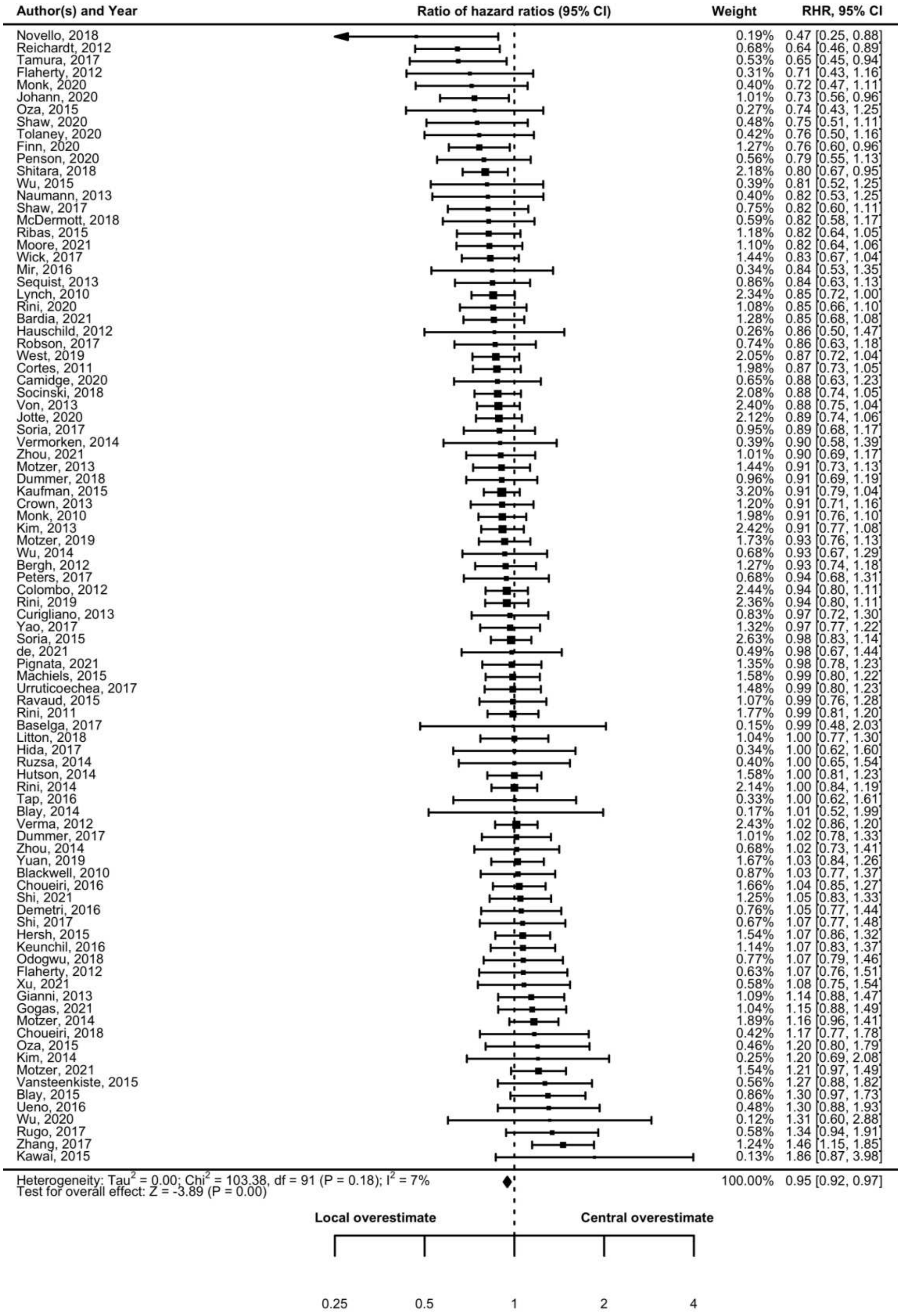

Funada S, *et al. BMJ EBM* 2023; 28:373–383. doi: 10.1136/bmjebm-2023-112332

Supplementary Figure 4c. Comparison of treatment effect estimates (Hazard Ratio) between central reviewers and local investigators. Sensitivity analysis (ρ = 0.75).

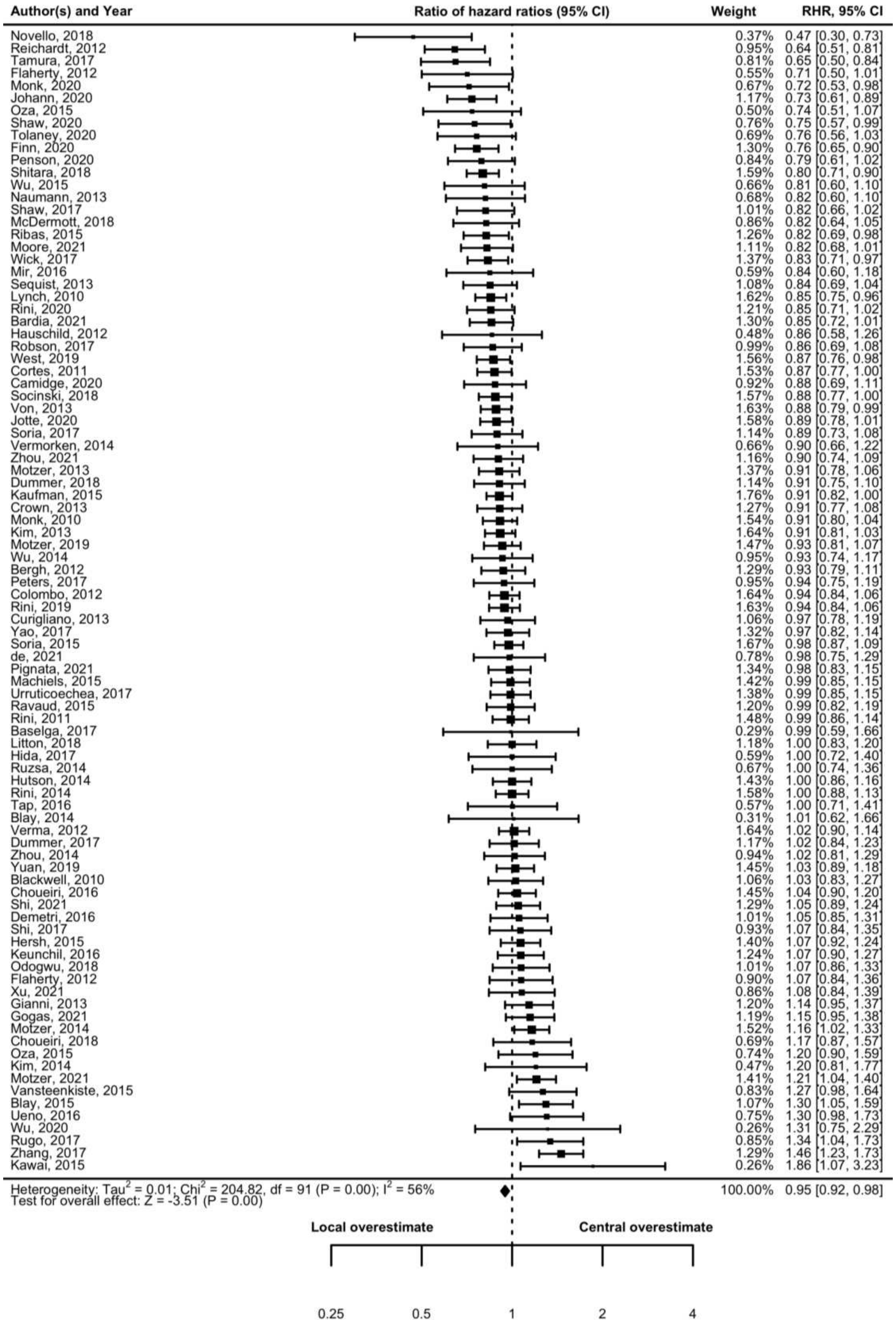

Funada S, *et al. BMJ EBM* 2023; 28:373–383. doi: 10.1136/bmjebm-2023-112332

Supplementary Figure 4d. Comparison of treatment effect estimates (Hazard Ratio) between central reviewers and local investigators. Sensitivity analysis (ρ = 0.95).

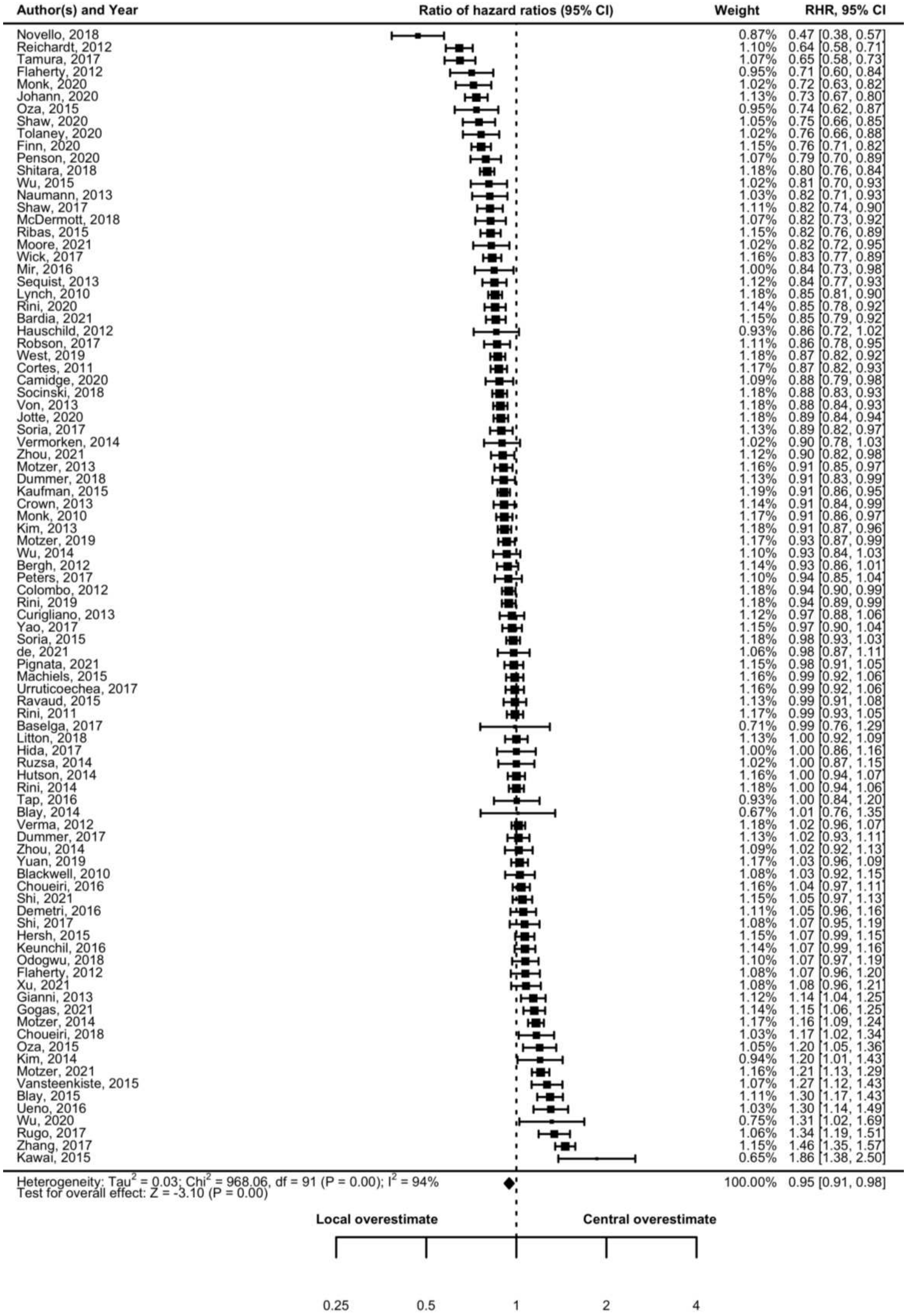

7

Supplementary Figure 5a. Comparison of treatment effect estimates (Odds Ratio) between central reviewers and local investigators. Sensitivity analysis (ρ = 0.25).

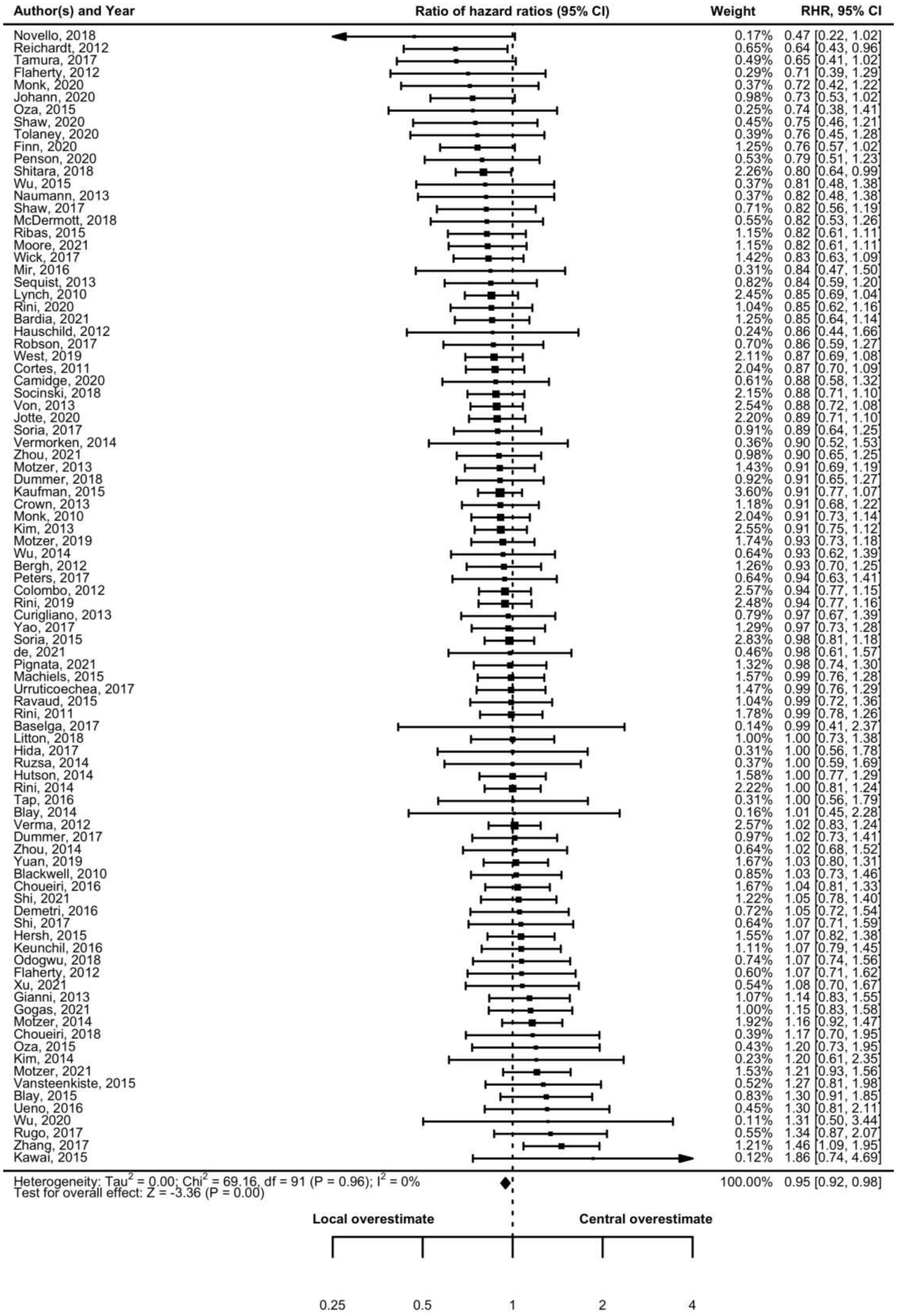

Funada S, *et al. BMJ EBM* 2023; 28:373–383. doi: 10.1136/bmjebm-2023-112332

Supplementary Figure 5b. Comparison of treatment effect estimates (Odds Ratio) between central reviewers and local investigators. Sensitivity analysis (ρ = 0.50).

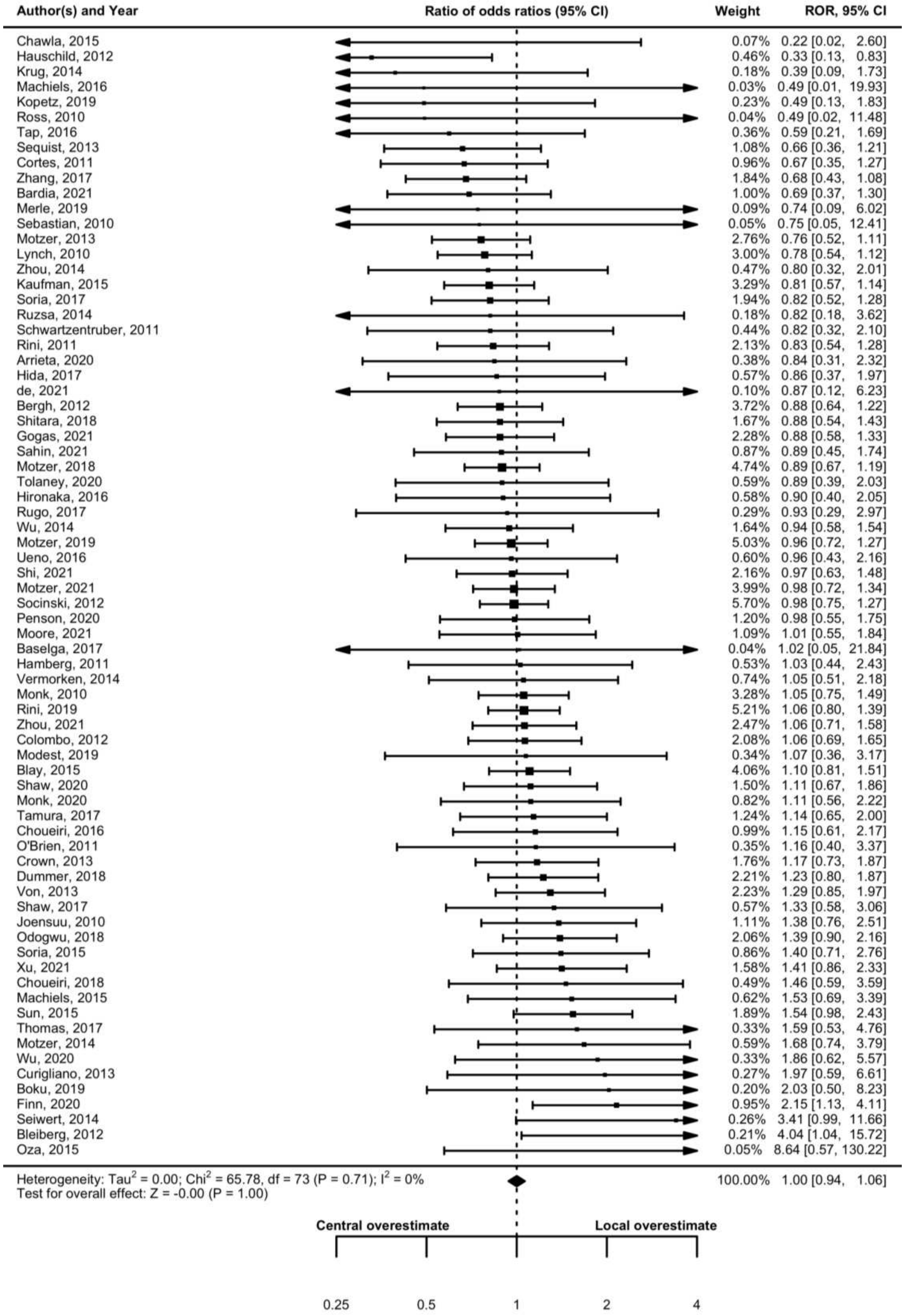

Supplementary Figure 5c. Comparison of treatment effect estimates (Odds Ratio) between central reviewers and local investigators. Sensitivity analysis (ρ = 0.75).

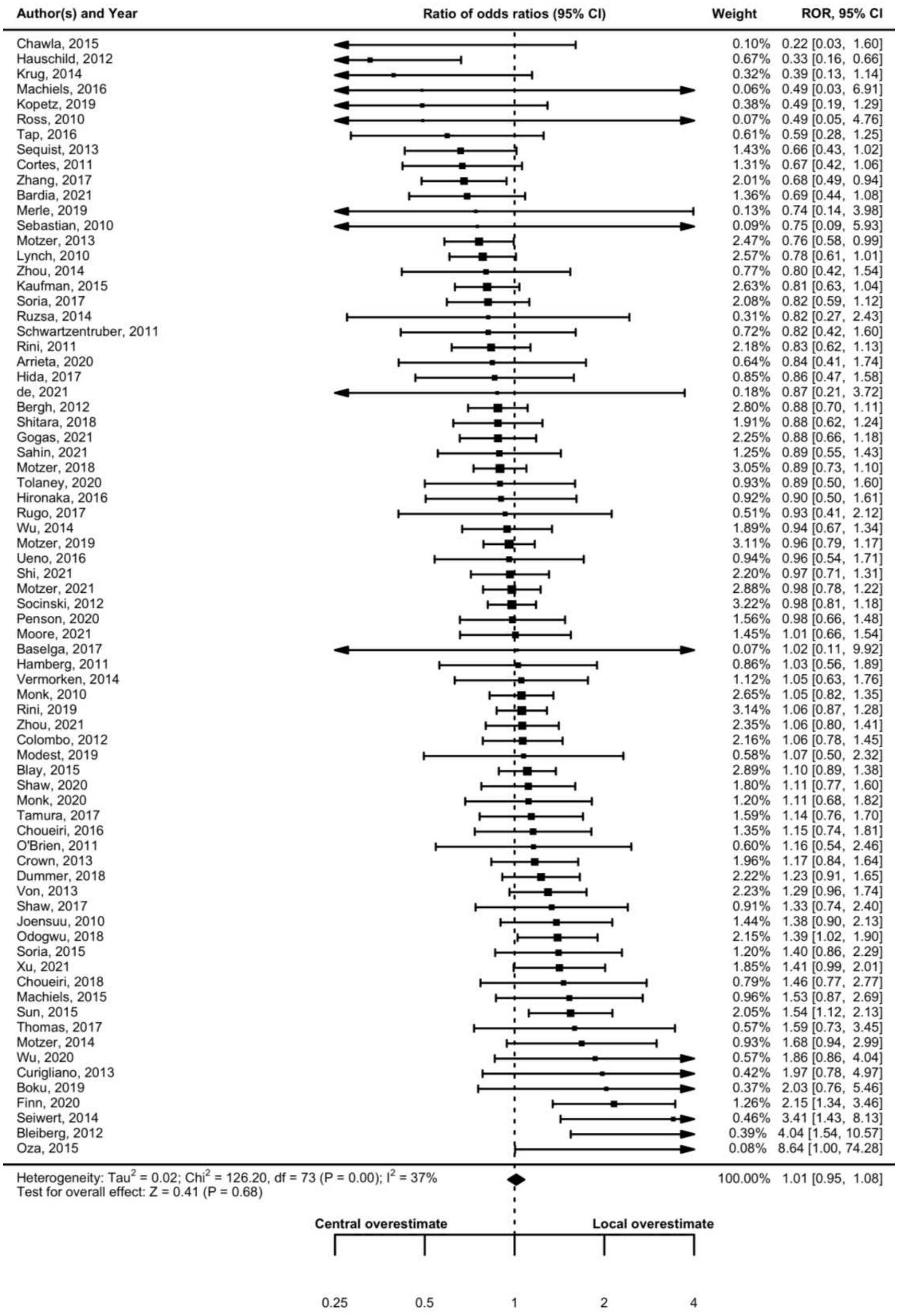

Funada S, *et al. BMJ EBM* 2023; 28:373–383. doi: 10.1136/bmjebm-2023-112332

Supplementary Figure 5d. Comparison of treatment effect estimates (Odds Ratio) between central reviewers and local investigators. Sensitivity analysis (ρ = 0.95).

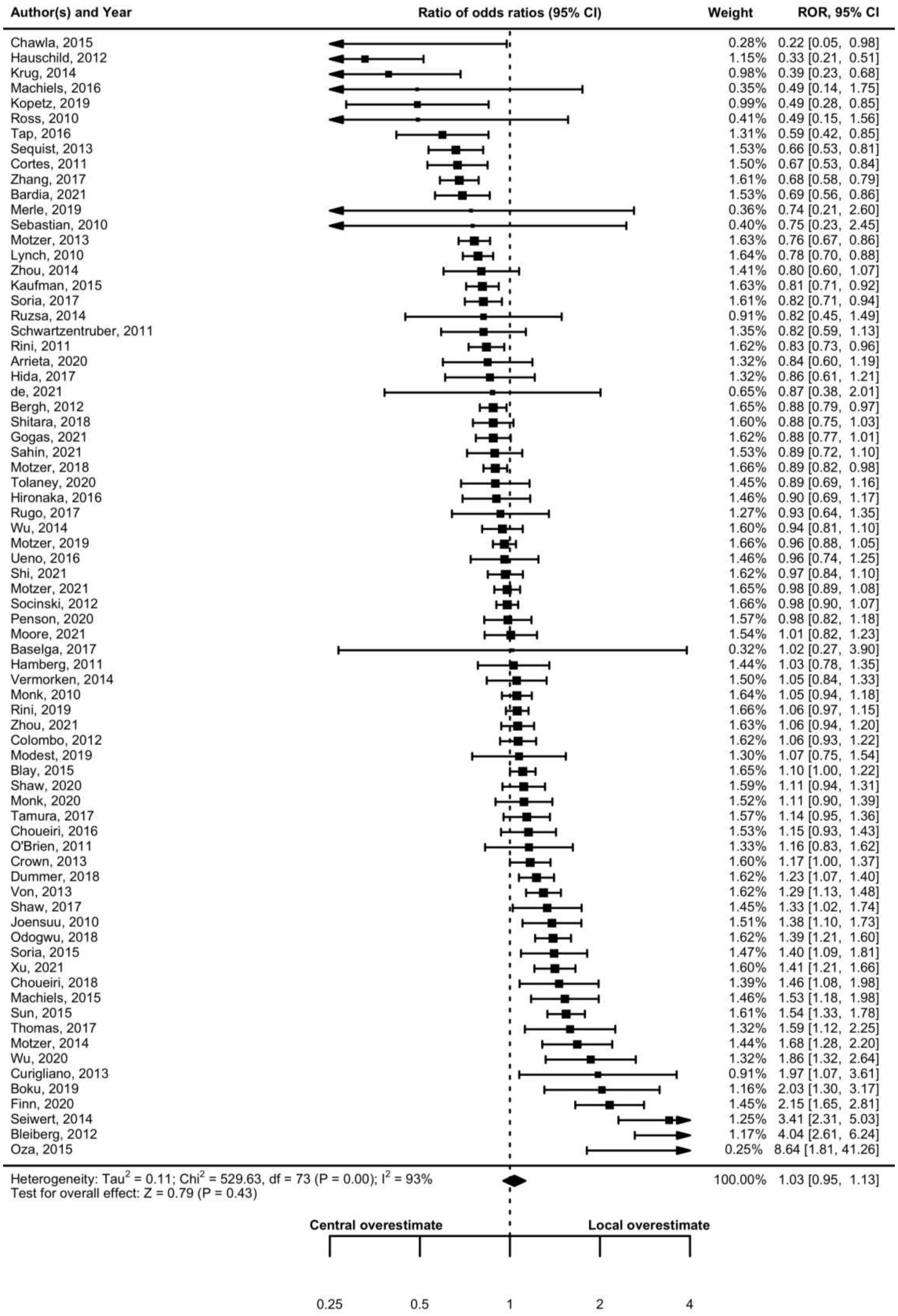

Funada S, *et al. BMJ EBM* 2023; 28:373–383. doi: 10.1136/bmjebm-2023-112332

Supplementary Figure 6a. Comparison of treatment effect estimates (Hazard Ratio) between central reviewers and local investigators. Subgroup analysis: trial phase.

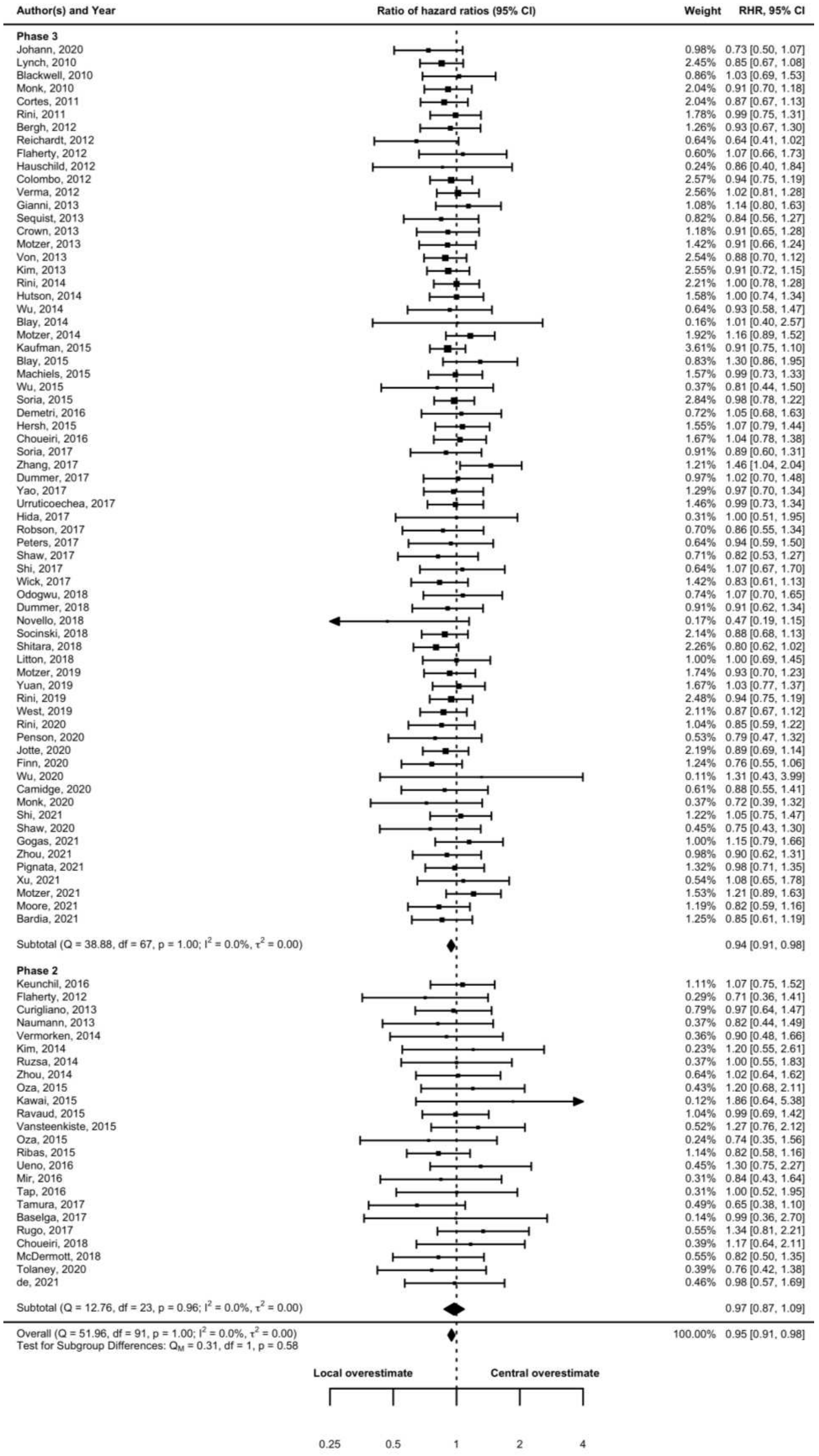

Funada S, et al. BMJ EBM 2023; 28:373–383. doi: 10.1136/bmjebm-2023-112332

Supplementary Figure 6b. Comparison of treatment effect estimates (Odds Ratio) between central reviewers and local investigators. Subgroup analysis: trial phase.

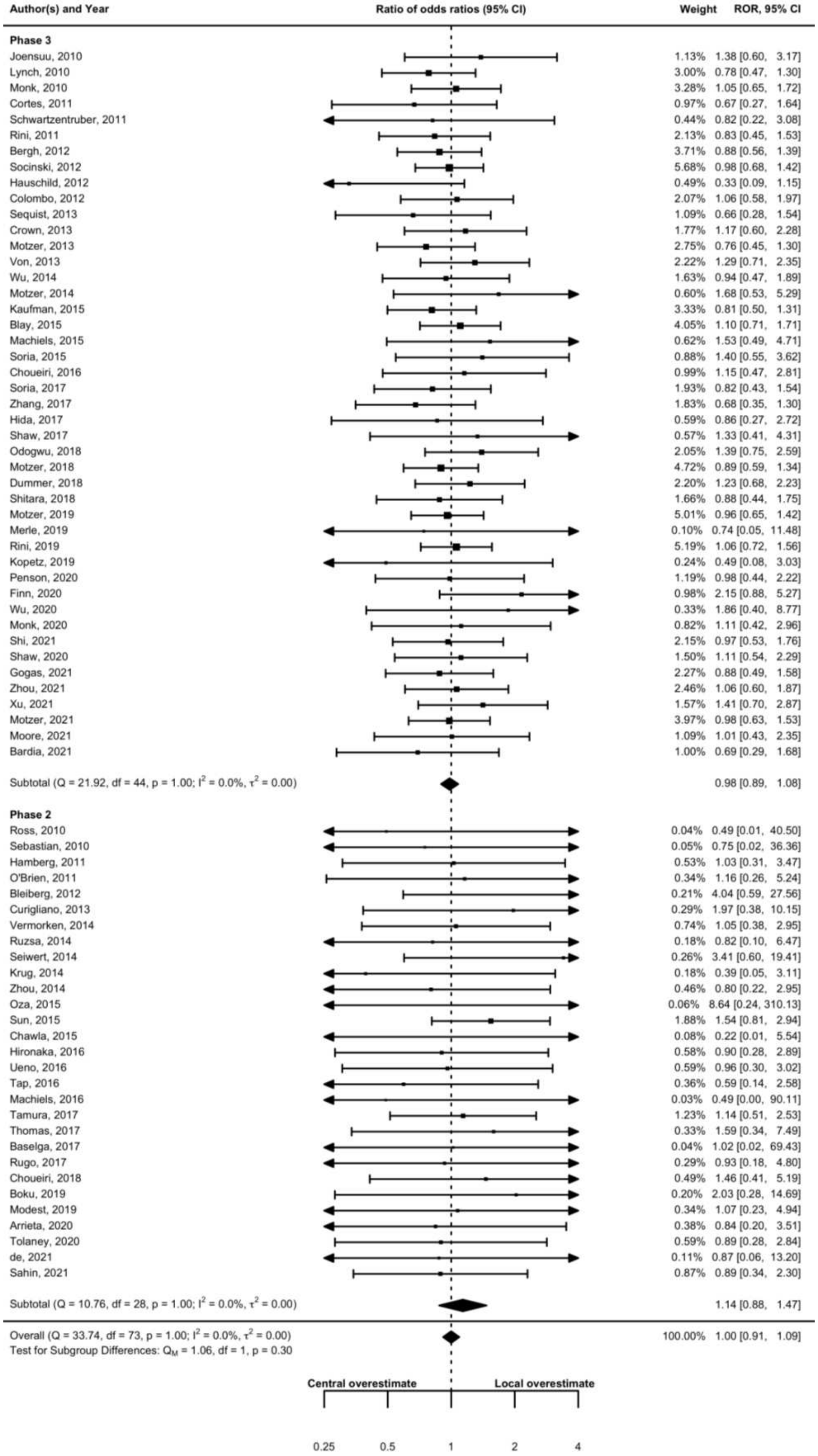

Funada S, *et al. BMJ EBM* 2023; 28:373–383. doi: 10.1136/bmjebm-2023-112332

Supplementary Figure 7a. Comparison of treatment effect estimates (Hazard Ratio) between central reviewers and local investigators. Subgroup analysis: cancer type.

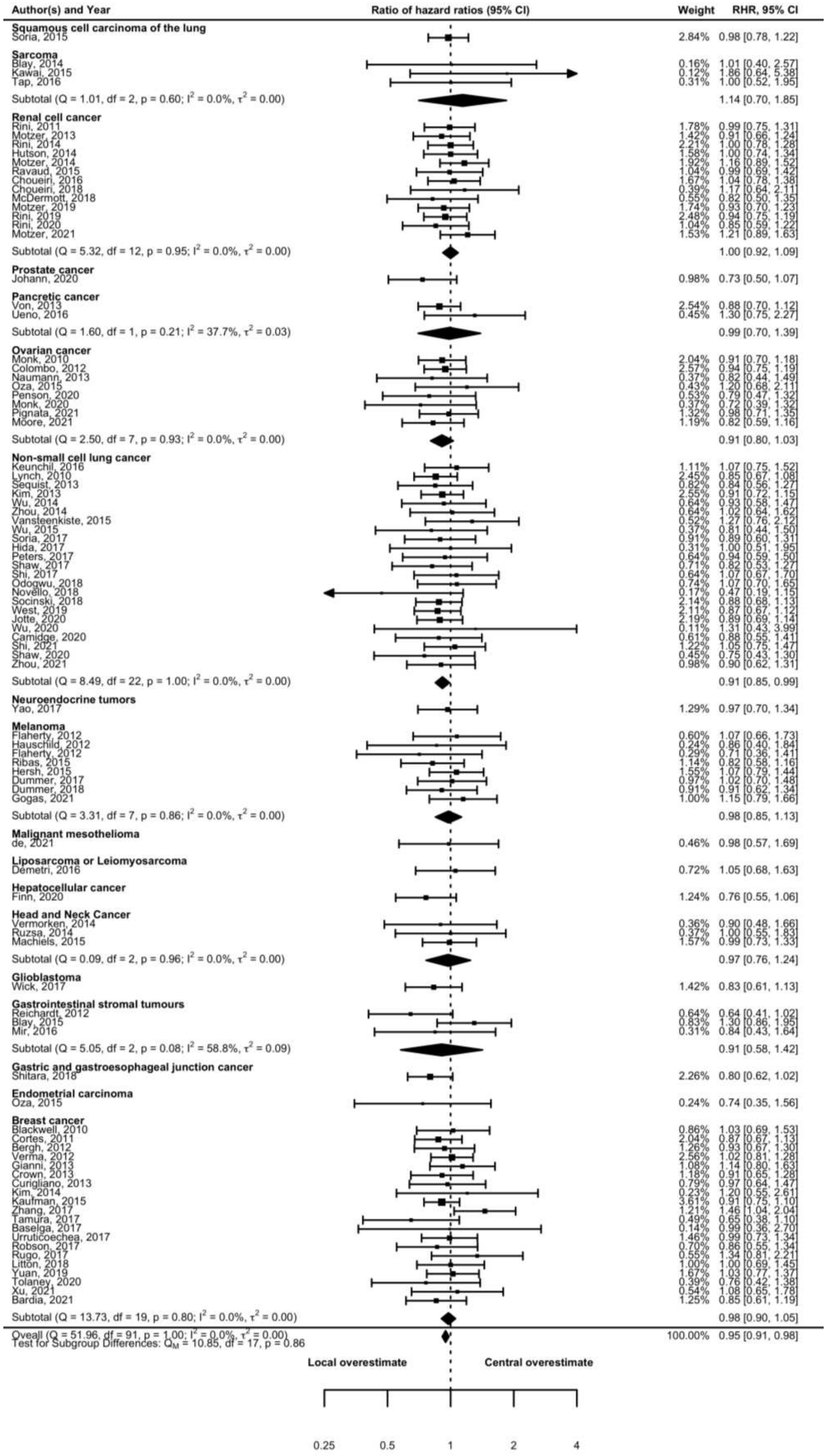

Supplementary Figure 7b. Comparison of treatment effect estimates (Odds Ratio) between central reviewers and local investigators. Subgroup analysis: cancer type.

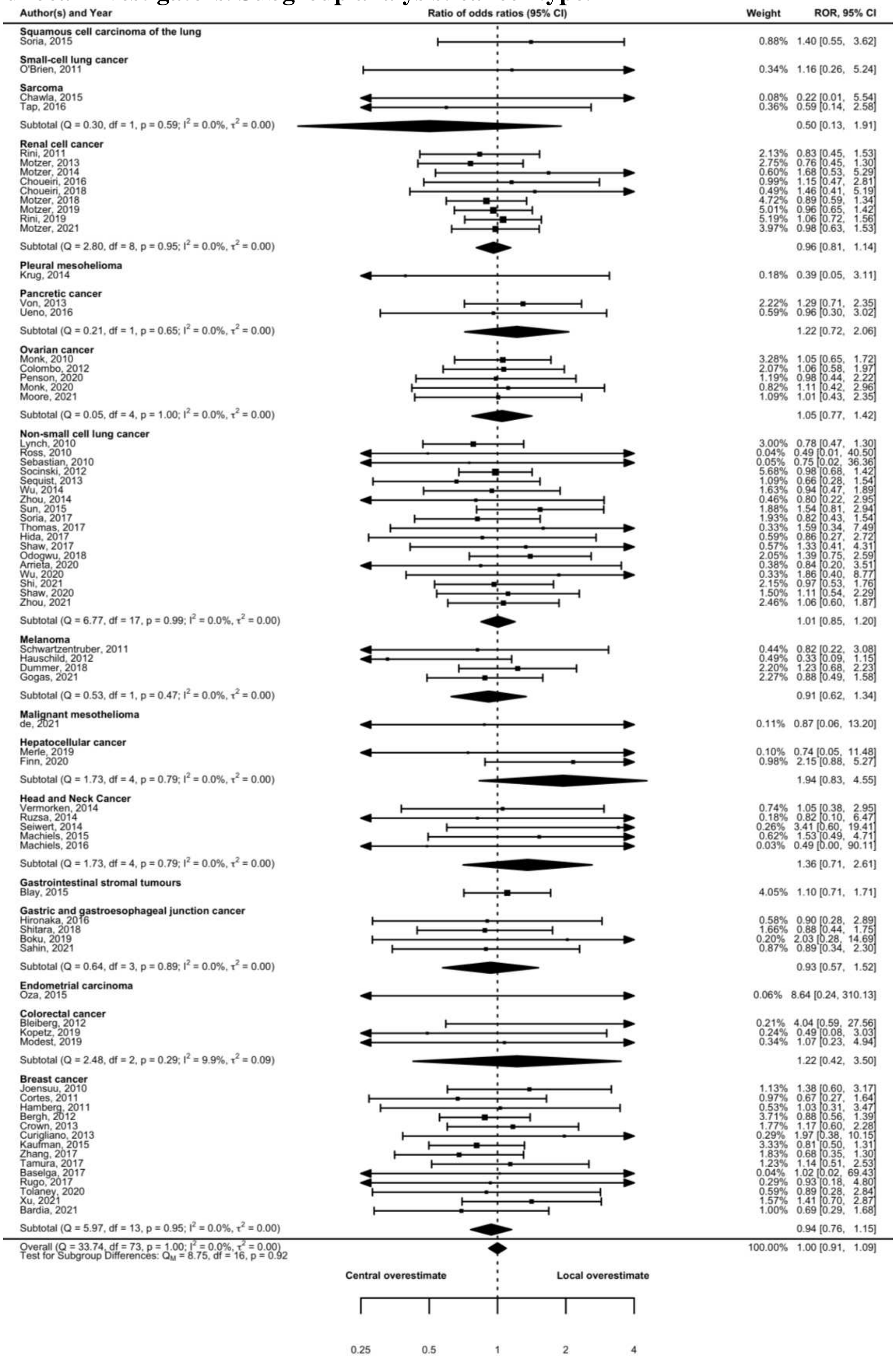

Funada S, *et al. BMJ EBM* 2023; 28:373–383. doi: 10.1136/bmjebm-2023-112332

Supplementary Figure 8a. Comparison of treatment effect estimates (Hazard Ratio) between central reviewers and local investigators. Subgroup analysis: drug classification.

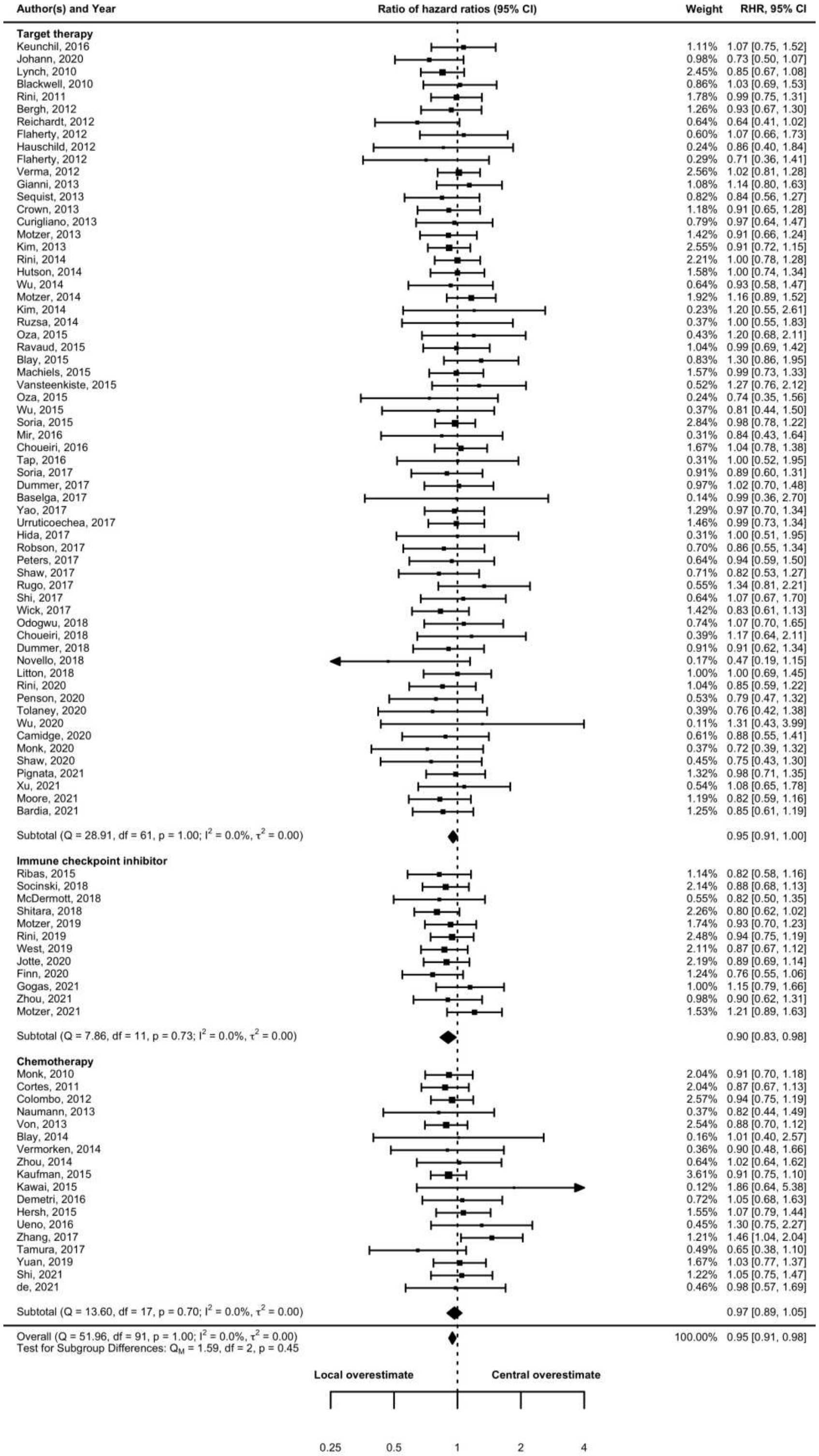

Funada S, *et al. BMJ EBM* 2023; 28:373–383. doi: 10.1136/bmjebm-2023-112332

Supplementary Figure 8b. Comparison of treatment effect estimates (Odds Ratio) between central reviewers and local investigators. Subgroup analysis: drug classification.

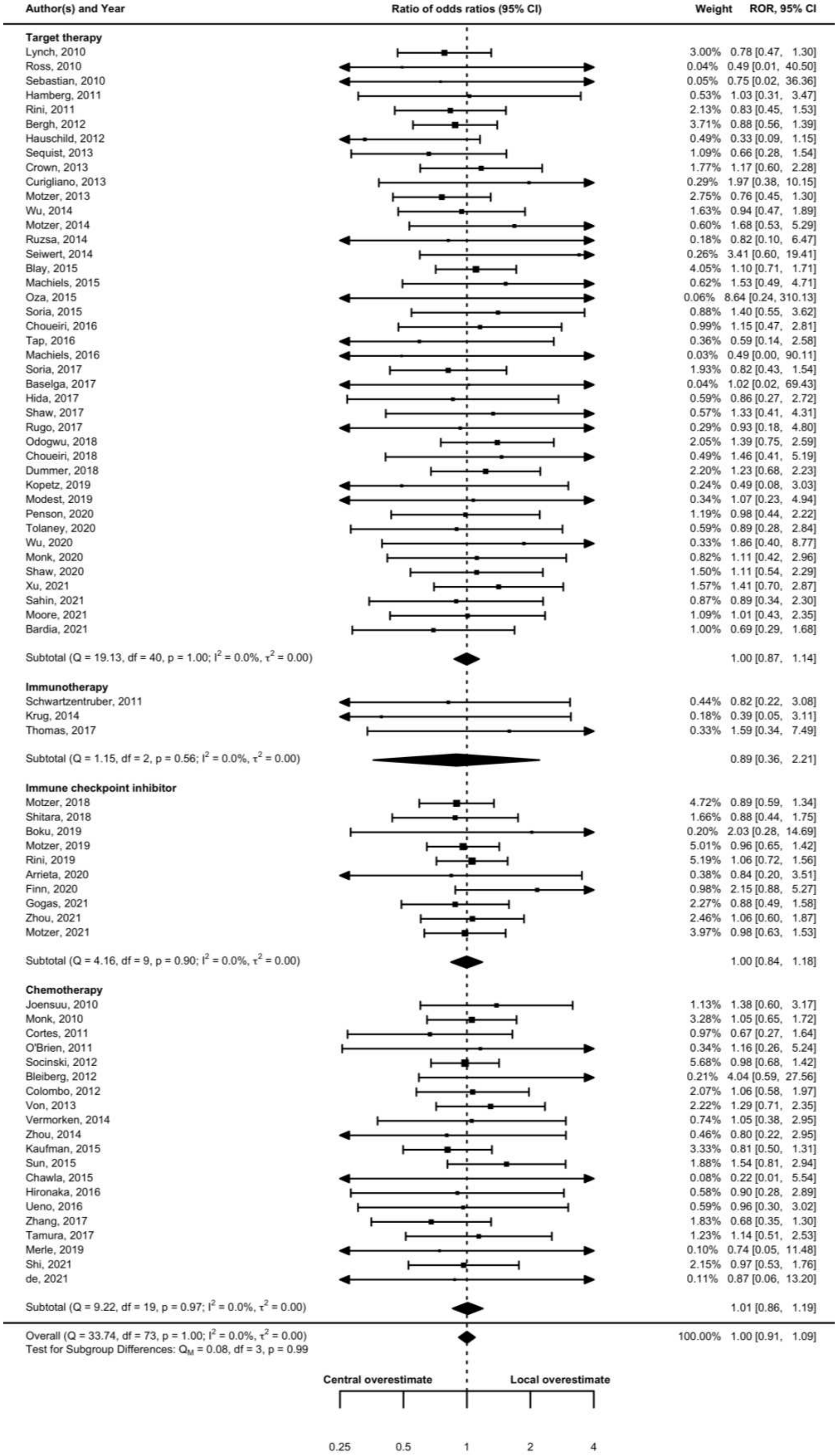

Funada S, *et al. BMJ EBM* 2023; 28:373–383. doi: 10.1136/bmjebm-2023-112332

Supplementary Table 1. Adjudicators of open-label trials of anticancer drugs (n = 1,197)

PFS	
Central + local	157 (13.1)
Only central	134 (11.2)
Only local	291 (24.3)
Unclear	528 (44.1)
No PFS	87 (7.3)
ORR	
Central + local	141 (11.8)
Only central	158 (13.2)
Only local	301 (25.2)
Unclear	536 (44.8)
No ORR	61 (5.1)

Abbreviations: ORR, objective response rate; PFS, progression-free survival.

Funada S, *et al. BMJ EBM* 2023; 28:373–383. doi: 10.1136/bmjebm-2023-112332

Supplementary Table 2. Effect of correlation between central and local adjudications (sensitivity analysis).

PFS				
ρ	RHR (95%CI)	Tau^2	I^2	P value for heterogeneity
0	0.95 (0.91 to 0.98)	0.00	0%	>0.99
0.25	0.95 (0.92 to 0.98)	0.00	0%	0.96
0.50	0.95 (0.92 to 0.97)	0.00	7.0%	0.18
0.75	0.95 (0.92 to 0.98)	0.01	56.0%	< .0001
0.95	0.95 (0.91 to 0.98)	0.03	94.0%	< .0001
ORR				
ρ	ROR (95%CI)	Tau^2	I^2	P value for heterogeneity
0	1.00 (0.91 to 1.09)	0.00	0%	>0.99
0.25	1.00 (0.93 to 1.08)	0.00	0%	>0.99
0.50	1.00 (0.94 to 1.06)	0.00	0%	0.71
0.75	1.01 (0.95 to 1.08)	0.02	37.0%	< .0001
0.95	1.03 (0.95 to 1.13)	0.11	93.0%	< .0001

Abbreviations: ORR, objective response rate; PFS, progression-free survival; RHR, ratios of hazard ratio; ROR, ratios of odds ratio.

A ρ is correlation coefficients between central and local adjudications in each trial.

A ρ of 0 indicates no dependency and a r of 0.95 indicates almost complete dependency.

Funada S, *et al. BMJ EBM* 2023; 28:373–383. doi: 10.1136/bmjebm-2023-112332
